# Lymph node migratory dendritic cells modulate HIV-1 transcription through PD-1 engagement

**DOI:** 10.1371/journal.ppat.1007918

**Published:** 2019-07-22

**Authors:** Riddhima Banga, Caterina Rebecchini, Francesco Andrea Procopio, Alessandra Noto, Olivia Munoz, Kalliopi Ioannidou, Craig Fenwick, Khalid Ohmiti, Matthias Cavassini, Jean-Marc Corpataux, Laurence de Leval, Giuseppe Pantaleo, Matthieu Perreau

**Affiliations:** 1 Service of Immunology and Allergy, Lausanne University Hospital, University of Lausanne, Lausanne, Switzerland; 2 Institute of Pathology, Lausanne University Hospital, University of Lausanne, Lausanne, Switzerland; 3 Service of Infectious Diseases, Lausanne University Hospital, University of Lausanne, Lausanne, Switzerland; 4 Service of Vascular Surgery, Lausanne University Hospital, University of Lausanne, Lausanne, Switzerland; 5 Swiss Vaccine Research Institute, Lausanne University Hospital, University of Lausanne, Lausanne, Switzerland; Emory University, UNITED STATES

## Abstract

T-follicular helper (Tfh) cells, co-expressing PD-1 and TIGIT, serve as a major cell reservoir for HIV-1 and are responsible for active and persistent HIV-1 transcription after prolonged antiretroviral therapy (ART). However, the precise mechanisms regulating HIV-1 transcription in lymph nodes (LNs) remain unclear. In the present study, we investigated the potential role of immune checkpoint (IC)/IC-Ligand (IC-L) interactions on HIV-1 transcription in LN-microenvironment. We show that PD-L1 (PD-1-ligand) and CD155 (TIGIT-ligand) are predominantly co-expressed on LN migratory (CD1c^high^CCR7^+^CD127^+^) dendritic cells (DCs), that locate predominantly in extra-follicular areas in ART treated individuals. We demonstrate that TCR-mediated HIV production is suppressed *in vitro* in the presence of recombinant PD-L1 or CD155 and, more importantly, when LN migratory DCs are co-cultured with PD-1^+^/Tfh cells. These results indicate that LN migratory DCs expressing IC-Ls may more efficiently restrict HIV-1 transcription in the extra-follicular areas and explain the persistence of HIV transcription in PD-1^+^/Tfh cells after prolonged ART within germinal centers.

## Author summary

Increasing number of evidences indicate that B-cell follicles might be anatomical sanctuaries for active transcription in both HIV/SIV viremic controllers and in ART treated aviremic HIV-infected individuals. While multiple mechanisms may be involved in the regulation of HIV transcription, recent studies suggested that immune checkpoint molecule (IC) signaling may contribute to maintain HIV-1 latency in infected CD4 T cells. These observations prompted us to investigate the involvement of IC/IC-L interactions in the regulation of HIV-1 transcription in lymph node (LN) tissues. In the present study, we show that T follicular helper (Tfh) cells predominantly co-expressed PD-1 and TIGIT, which were functionally active. An in-depth mass cytometry analysis revealed that PD-L1, PD-L2 (PD-1 ligands) and CD155 (TIGIT-ligand) were predominantly co-expressed on a specific LN dendritic cell (DC) subpopulation expressing markers of migratory DCs. We subsequently demonstrated that LN migratory DCs, locating predominantly in LN extra-follicular areas, could modulate HIV-1 transcription by a mechanism involving PD-L1/PD-1 interactions. Interestingly, the frequency of LN migratory DCs inversely correlated with HIV-1 transcription from LN memory CD4 T cells, suggesting that IC-L expressing migratory DCs might contribute to control HIV-1 transcription and maintain HIV-1 latency in extra-follicular areas. These findings represent a step forward in our understanding of potential mechanisms contributing to the regulation of HIV persistence in lymphoid tissues.

## Introduction

One of the major obstacles to HIV-1 eradication resides in the capacity of HIV-1 to rapidly establish a latent reservoir, transcriptionally silent, which is not susceptible to both the host immune response and cART [1–6]. Different cell lineages including CD4 T cells and monocytes/macrophages [7–9] may contribute to the HIV-1 reservoir. Central memory and transitional memory CD4 T cells serve as major cellular compartments of the latent HIV-1 reservoir in blood [6]. More recently, blood memory CD4 T cells with stem-cell like properties [10] or expressing CXCR3 and/or CCR6 were also shown to contain latently HIV-infected cells [11–13]. However, blood contains only 2% of the total lymphocytes that reside predominantly within lymphoid organs[14], and lymphocyte populations within the lymphoid tissues may be phenotypically and functionally distinct from those in blood [15] with differential cell composition and extensive cell heterogeneity regarding T follicular helper (Tfh) cells [16]. Notably, previous studies have demonstrated that lymphoid organs are the major anatomic site for HIV infection, production and spreading and that high concentration of virus particles and CD4 T cells with active virus replication were predominantly restricted to germinal centers (GCs) in viremic individuals [17–19].

Interestingly, recent studies have underscored that while HIV or SIV DNA containing CD4 T cells were consistently detected in cells located in lymph node (LN) follicular and extra-follicular areas in long-term cART treated individuals [11, 20, 21], HIV or SIV RNA detection were mainly restricted to CD4 T cells in LN GC areas of HIV viremic controllers [22], SIV-infected elite controller macaques [23] or cART-treated aviremic HIV-infected individuals [24]. Of note, Tfh cells represent the major cellular compartment for HIV production and replication in viremic individuals [25, 26] and the major CD4 T cell population for persistent HIV-1 transcription in long-term treated individuals [24] as compared to any other blood or LN memory CD4 T cell populations. The limited access of cytotoxic CD8 T cells to GCs[23], the sub-optimal antiretroviral drug penetration in lymphoid tissues [27] and the higher level of activation of Tfh cells [26] are possible explanations for lymphoid organs serving as primary anatomic sites for HIV infection and persistence.

Multiple cellular mechanisms are involved in the establishment and the maintenance of HIV-1 latency including 1) epigenetic silencing induced by histone deacetylation and DNA methylation [28, 29], 2) limiting cellular levels of the essential Tat cofactor P-TEFb and the transcription initiation factors NF-kB and NFAT [30] and 3) condensed chromatin at the viral long terminal repeat [31]. However, under certain circumstances, HIV-1 transcription/production might be reactivated. The parameters associated with HIV-1 reactivation include T-cell receptor (TCR)-mediated signaling *via* NF-κB [29, 32], cytokine and chemokine stimulations [33] or epigenetic DNA modifications such as acetylation and methylation [34]. Interestingly, Fromentin *et al*. showed that the expression of immune checkpoint molecules (ICs) such as PD-1, LAG-3 and TIGIT on memory CD4 T cells was associated with HIV-infected cells in distinct blood memory CD4 T cell subsets during ART [35], suggesting that IC signaling may contribute to maintain HIV-1 latency in HIV-1 infected memory CD4 T cells [36, 37].

Given that previous studies have underscored the involvement of IC/IC-L interactions in the functional impairment of Tfh cells in viremic HIV-infected individuals [38–40], we hypothesized that IC/IC-ligand (IC-L) interactions may contribute to modulate HIV latency/virus reactivation in the LN microenvironment. In the present study, we therefore investigated 1) the expression and distribution of ICs and IC-Ls in blood and LN mononuclear cells isolated from cART treated aviremic, viremic and HIV-uninfected subjects using mass cytometry and *in situ* in lymph node compartments (germinal centers and extra-follicular zones) by immunohistochemistry, and 2) the impact of IC/IC-L interactions on TCR-mediated T-cell proliferation and HIV-1 transcription/production.

We demonstrate that PD-1 and TIGIT, the two major ICs expressed on Tfh cells *ex vivo*, are functionally active and regulate TCR-mediated HIV-1 transcription and production *in vitro*. However, PD-L1 (PD-1-ligand) and CD155 (TIGIT-ligand) were predominantly co-expressed on LN migratory (CD1c^high^CCR7^+^CD127^+^) dendritic cells (DCs) which located mainly in the extra-follicular areas of cART treated subjects. These findings suggest that the strength of the inhibitory signal resulting from the IC/IC-L interactions might be selectively reduced in GCs of ART treated subjects, thus creating a microenvironment less constrained for cell activation and HIV transcription. In support of this hypothesis, we demonstrate that LN migratory DCs *via* IC/IC-L interactions modulate TCR-mediated HIV-1 reactivation and production from LN PD-1^+^/Tfh cells of cART treated HIV-infected individuals and that the levels of HIV-1 transcription in LN memory CD4 T cells correlated with the reduced frequency of LN migratory DCs.

These findings indicate that the IC-L-mediated modulation of HIV-1 transcription in treated subjects is more efficient in extra-follicular areas and underscore that an imbalance in IC/IC-L interactions is a novel mechanism contributing to HIV-1 persistence in LNs.

## Results

### PD-1 and TIGIT are the two major ICs expressed on blood and LN memory CD4 T-cell populations including T follicular helper cells

**Fig 1:**
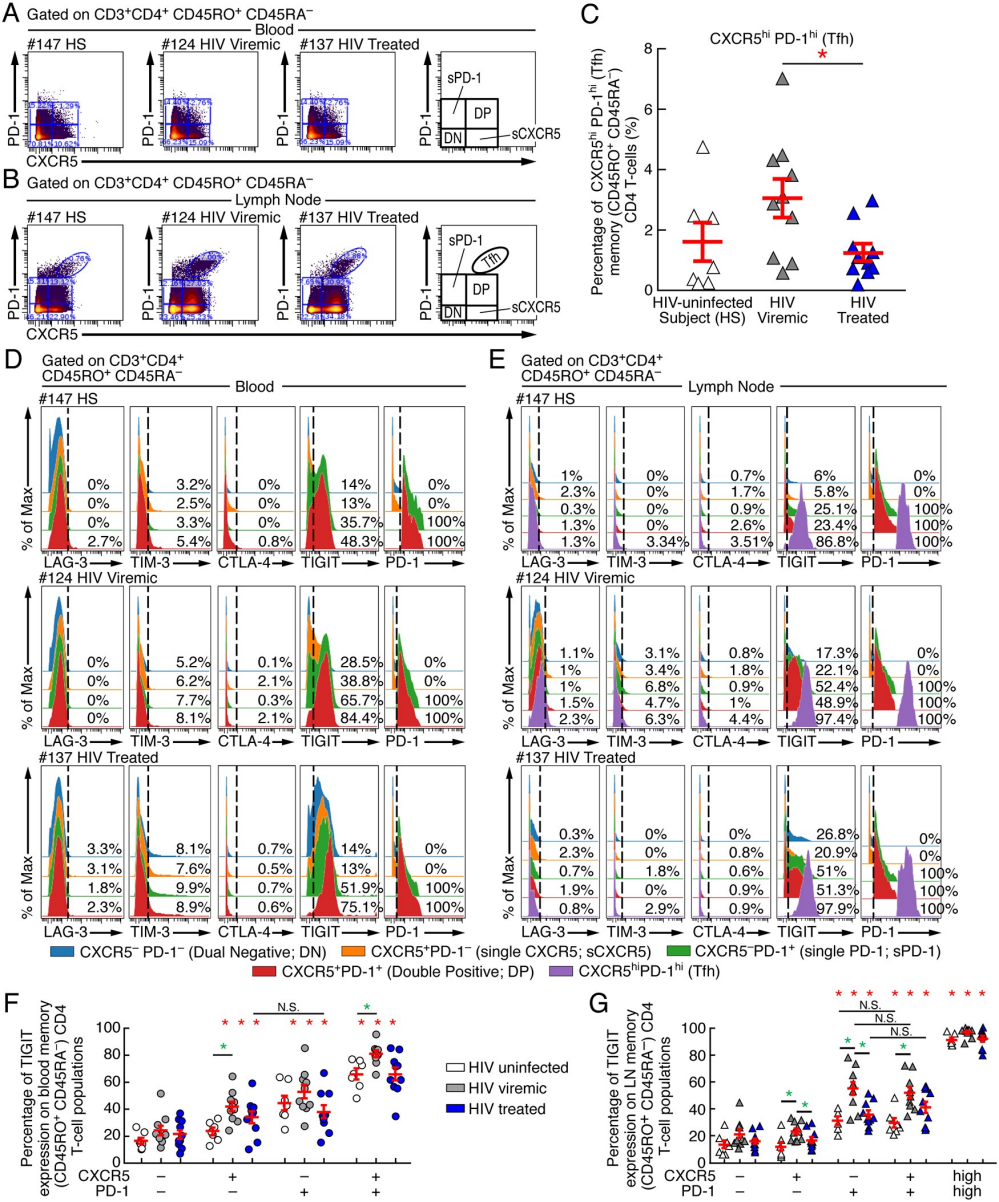
PD-1 and TIGIT are the two major ICs expressed on blood and LN memory CD4 T-cell populations including T-follicular helper cells. Representative mass cytometry profile of blood **(A)** and LN **(B)** memory (CD45RA^-^) CD4 T-cell populations expressing CXCR5 and/or PD-1 of representative HIV-uninfected (#147), viremic (#124) and aviremic ART treated HIV-infected individual (#137). **(C)** Frequencies of LN CXCR5^high^PD-1^high^ (Tfh) memory (CD45RA^-^) CD4 T cells in HIV-uninfected (N = 7), viremic (N = 10) and aviremic ART treated HIV-infected individuals (N = 10). **(D-E)** Level of expression of LAG-3, TIM-3, CTLA-4, TIGIT and PD-1 on CXCR5^−^PD-1^−^ (DN), CXCR5^−^PD-1^+^ (single PD-1; sPD-1), CXCR5^+^PD-1− (single CXCR5; sCXCR5), CXCR5^+^PD-1^+^ (Double positive; DP) and CXCR5^high^PD-1^high^ (Tfh) memory CD4 T cell populations from blood **(D)** and LN **(E)** of one representative HIV-uninfected (#147), viremic (#124) and aviremic ART treated HIV-infected individual (#137). The dashed line corresponds to the cut-off of positivity of IC-molecule expression which was based on the expression on naïve (CD45RO^-^CD45RA^+^) CD4 T cells **(D-E)**. Percentage of TIGIT expression on blood **(F)** and LN **(G)** memory (CD45RA^-^) CD4 T-cell populations of HIV-uninfected (N = 7), viremic (N = 10) and aviremic ART treated HIV-infected individuals (N = 10). White symbols correspond to HIV-uninfected subjects, grey symbols corresponds to HIV-1 viremic individuals and blue symbols correspond to HIV-infected aviremic ART treated individuals **(C, F-G)**. Red bars correspond to mean ± SEM **(C, F-G)**. CD4 T-cell populations were color-coded **(D-E)**. Red stars indicate statistical significance (* = *P*<0.05) for intra-group comparisons whereas green stars indicate statistical significance (* = *P*<0.05) for inter-population comparisons. Statistical significance (*P* values) was obtained using one-way ANOVA (Kruskal-Wallis test) followed by Mann Whitney test (intra-group comparisons) or Wilcoxon Matched-pairs two-tailed Signed Rank test (inter-population comparisons).

We simultaneously collected blood and LN from 10 viremic and 10 cART treated aviremic HIV-1 infected individuals and 7 HIV-uninfected subjects. Mononuclear cells isolated from blood and LNs were then stained with a mass cytometry panel encompassing 38 markers including cell lineage markers, ICs and IC-ligands, *i*.*e*. PD-1, CTLA-4, LAG-3, TIM-3, TIGIT, PD-L1, PD-L2 and CD155. The extracellular expression of CTLA-4, LAG-3, TIM-3 and TIGIT was assessed in blood and LN memory CD4 T-cell populations identified on the basis of the expression of PD-1 and/or CXCR5, *i*.*e*. CXCR5^-^PD-1^-^, CXCR5^+^PD-1^-^, CXCR5^-^PD-1^+^, CXCR5^int^PD-1^int^ and CXCR5^high^PD-1^high^ CD4 T cells (Fig 1A and 1B). Of note, CXCR5^high^PD-1^high^ CD4 T cells correspond to LN Tfh cells (Fig 1B). Consistent with a previous study [24, 26], LN Tfh cells of viremic untreated HIV-1 infected individuals were increased as compared to HIV-uninfected subjects and their percentage dropped after prolonged cART to levels observed in HIV-uninfected subjects (*P*<0.05) (Fig 1C).

Co-inhibitory molecule expression was determined extracellularly and the gating strategy defining the positivity of co-inhibitory molecules expression was set on naïve (CD45RA^+^CD45RO^-^) CD4 T cells for all co-inhibitory molecules tested (Fig 1D and 1E). The representative examples and cumulative data showed that PD-1 and TIGIT were the two major ICs expressed in CD4 T-cell populations of HIV-uninfected, viremic and aviremic cART treated HIV-1 infected individuals (Fig 1D–1G and S1 Fig). Interestingly, more than 90% of Tfh cells expressed TIGIT, while TIM-3, LAG-3 and/or CTLA-4 were expressed in less than 5% of Tfh cells (Fig 1D–1G and S1 Fig). Notably, TIGIT was also expressed in CXCR5^-^PD-1^-^, CXCR5^+^PD-1^-^, CXCR5^-^PD-1^+^ and CXCR5^int^PD-1^int^ CD4 T-cell populations isolated from blood and LN of HIV-uninfected, viremic and aviremic ART treated HIV-1 infected individuals, while TIM-3, LAG-3 and/or CTLA-4 were expressed in less than 10% of blood and LN memory CD4 T-cell populations (Fig 1D–1G and S1 Fig).

### PD-L1, PD-L2 and CD155 are predominantly expressed on lymph node migratory (CD1c^high^CCR7^+^CD127^+^) dendritic cells

We subsequently assessed the expression of PD-1 ligands, *i*.*e*. PD-L1, PD-L2 and TIGIT ligand, *i*.*e*. CD155, in blood and LN mononuclear cells including blood monocytes (CD14^+^), blood and LN DCs and various blood and LN B-cell populations, including LN GC B cells (CD19^+^IgD^-^CD38^high^CD10^+^) (S2 Fig). The gating strategy defining the positivity of IC-L expression was set on naïve (CD45RA^+^CD45RO^-^) CD4 T cells (S3 Fig).

**Fig 2:**
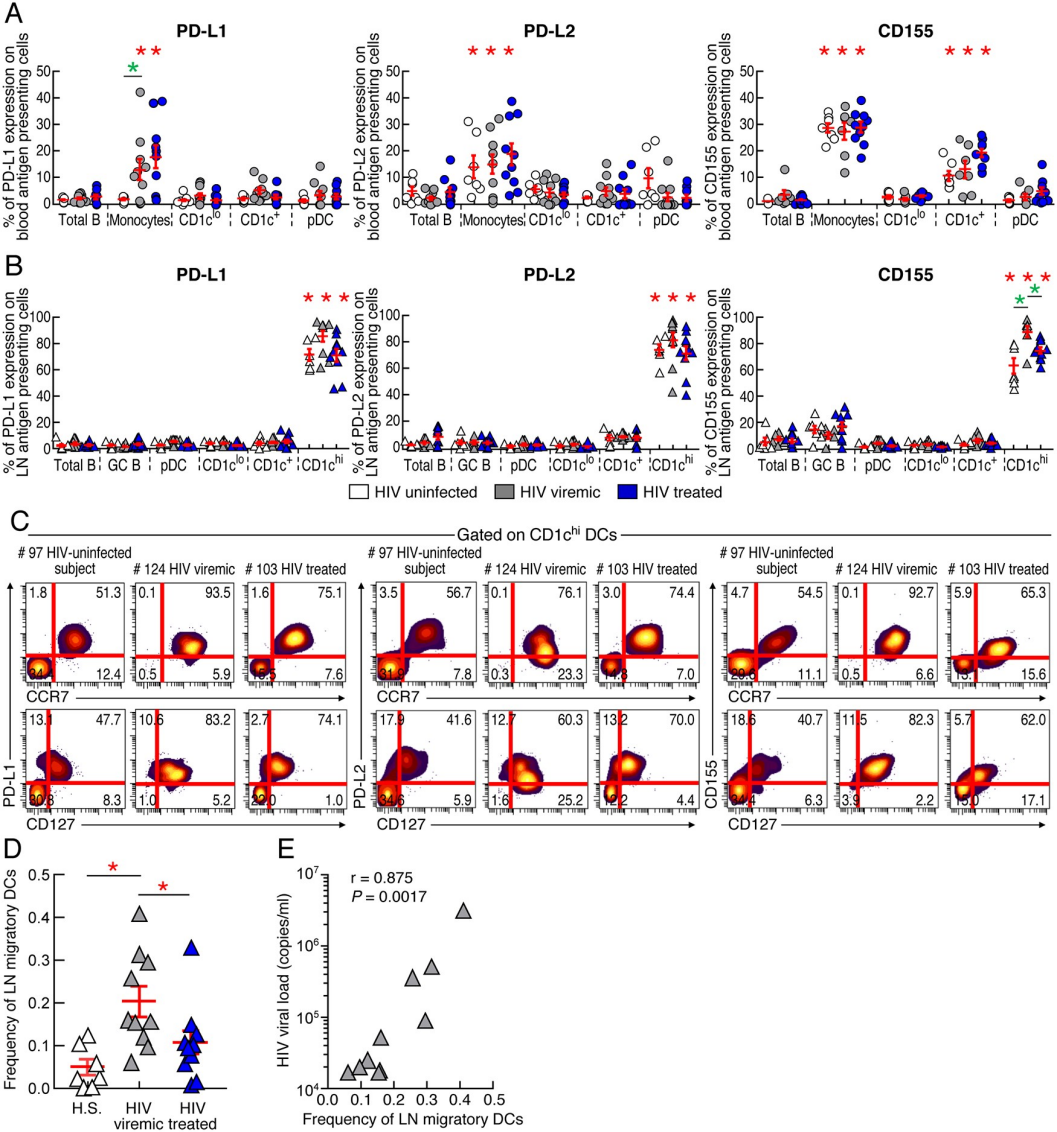
PD-L1, PD-L2 and CD155 are predominantly expressed on blood monocytes and lymph node migratory dendritic cells. **(A)** Percentage of PD-L1, PD-L2 or CD155 expression on blood **(A)** or LN **(B)** mononuclear-cell populations of HIV-uninfected (N = 7), viremic (N = 10) and aviremic ART treated HIV-infected individuals (N = 10). **(C)** Levels of IC-L expression on LN CD1c^high^ DCs expressing CCR7 and/or CD127 of a representative HIV-uninfected (#97), viremic (#124) and aviremic ART treated HIV-infected individual (#103). **(D)** Frequencies of LN migratory (CD1c^high^CCR7^+^CD127^+^) DCs in HIV-uninfected (N = 7), viremic (N = 10) and aviremic ART treated HIV-infected individuals (N = 10). **(E)** Correlation between the levels of HIV viral load and the frequencies of LN migratory DCs in viremic HIV-infected individuals (N = 10). White symbols correspond to HIV-uninfected subjects, grey corresponds to HIV-1 viremic individuals and blue symbols correspond to HIV-infected aviremic ART treated individuals **(A-B, D-E)**. Blood cell populations are represented in circles **(A)**. LN cell populations are represented in triangles **(B, D-E)**. Red bars correspond to mean ± SEM **(A-B, D)**. Red stars indicate statistical significance (*P*<0.05) for intra-group comparisons **(A-B, D)** whereas green stars indicate statistical significance (*P*<0.05) for inter-population comparisons **(A-B)**. Statistical significance (*P* values) was obtained using one-way ANOVA (Kruskal-Wallis test) followed by Mann Whitney test (intra-group comparisons) or Wilcoxon Matched-pairs two-tailed Signed Rank test (inter-population comparisons) or using Spearman rank test for correlations **(E)**.

The representative examples and cumulative data indicated that in blood, PD-L1, PD-L2 and CD155 were predominantly expressed on monocytes and to a lower extent on type 2 conventional DCs (cDC2; HLA-DR^+^CD11c^+^CD1c^+^) and plasmacytoïd DCs (pDC; HLA-DR^+^CD11c^-^CD123^+^), while IC-Ls were poorly expressed on blood B-cell populations of HIV-uninfected, viremic and aviremic ART treated HIV-1 infected individuals (*P*<0.05) (S3 Fig and Fig 2A). Of note, no correlation was observed between the frequencies of IC-L expressing blood monocytes with HIV viral load in viremic HIV-infected individuals (S4 Fig). However, although not statistically significant, but a trend towards a negative correlation was observed between the frequencies of PD-L-expressing monocytes and the duration of ART in treated individuals (r = -0.58; *P* = 0.08) (S4 Fig), suggesting that the expression of PD-Ls on monocytes may require longer treatment period to normalize.

In LN, IC-Ls were significantly more expressed on CD1c^high^ DCs than on pDCs and LN B-cell populations, including GC B cells of HIV-uninfected, viremic and aviremic ART treated HIV-1 infected individuals (*P*<0.05) (S3 Fig and Fig 2B).

We then further explored the phenotype of IC-L expressing LN CD1c^high^ DCs. The representative examples and cumulative data indicate that IC-L expressing LN CD1c^high^ DCs co-expressed CCR7 and CD127 (Fig 2C and S5 Fig), markers of LN migratory DCs [41, 42]. Lymph node CD1c^high^CCR7^+^CD127^+^ DCs were therefore referred to as LN migratory DCs.

We next investigated the influence of HIV-1 infection and treatment initiation on the frequency of LN migratory DCs of untreated viremic and treated aviremic HIV-1 infected individuals. Of note, non-reactive LNs obtained from HIV-uninfected subjects were used as control. The cumulative data indicated that the frequencies of LN migratory DCs of viremic untreated HIV-1 infected individuals were increased as compared to HIV-uninfected subjects, and their percentage dropped after prolonged cART to levels observed in HIV-uninfected subjects (*P*<0.05) (Fig 2D). In addition, the frequency of both LN migratory DCs and PD-L1/L2 expressing LN migratory DCs of viremic untreated HIV-1 infected individuals directly correlated with HIV-1 viral load (r = 0.875 and *P* = 0.0017; r = 0.8997 and *P* = 0.0009; r = 0.8693 and *P* = 0.0019, respectively) (Fig 2E and S6 Fig), suggesting that quantitative changes in IC-L-expressing LN migratory DCs might be associated with different levels of HIV load. Of note, no statistically significant correlation was observed between LN migratory DC and Tfh frequency or between PD1 and PD-L1 mean signal intensity (MFI) in untreated viremic HIV-infected individuals (*P*>0.05) (S6 Fig).

### HIV-1 infection and cART treatment initiation influences PD-L1 expression and tissue distribution

We then determined whether active and persistent virus transcription detected in LN PD-1^+^/Tfh cells [24] may result from reduced IC/IC-L interactions in the GC areas of cART treated HIV-1 infected individuals. For this purpose, the percentage of cells expressing PD-1 and the proportion of PD-L1 positive tissue surface were determined in GC and extra-follicular areas of LN sections collected from untreated viremic and treated aviremic HIV-1 infected individuals using immunohistochemistry staining as previously described[24]. Of note, to determine whether the spatial distribution of PD-1 and PD-L1-expressing cells observed in viremic HIV-infected individuals was associated with non-specific immune activation/inflammation or was specifically associated with HIV infection/replication, LN sections collected from HIV-uninfected individuals suffering from lymphadenopathy (“reactive LNs”) were used as control.

**Fig 3:**
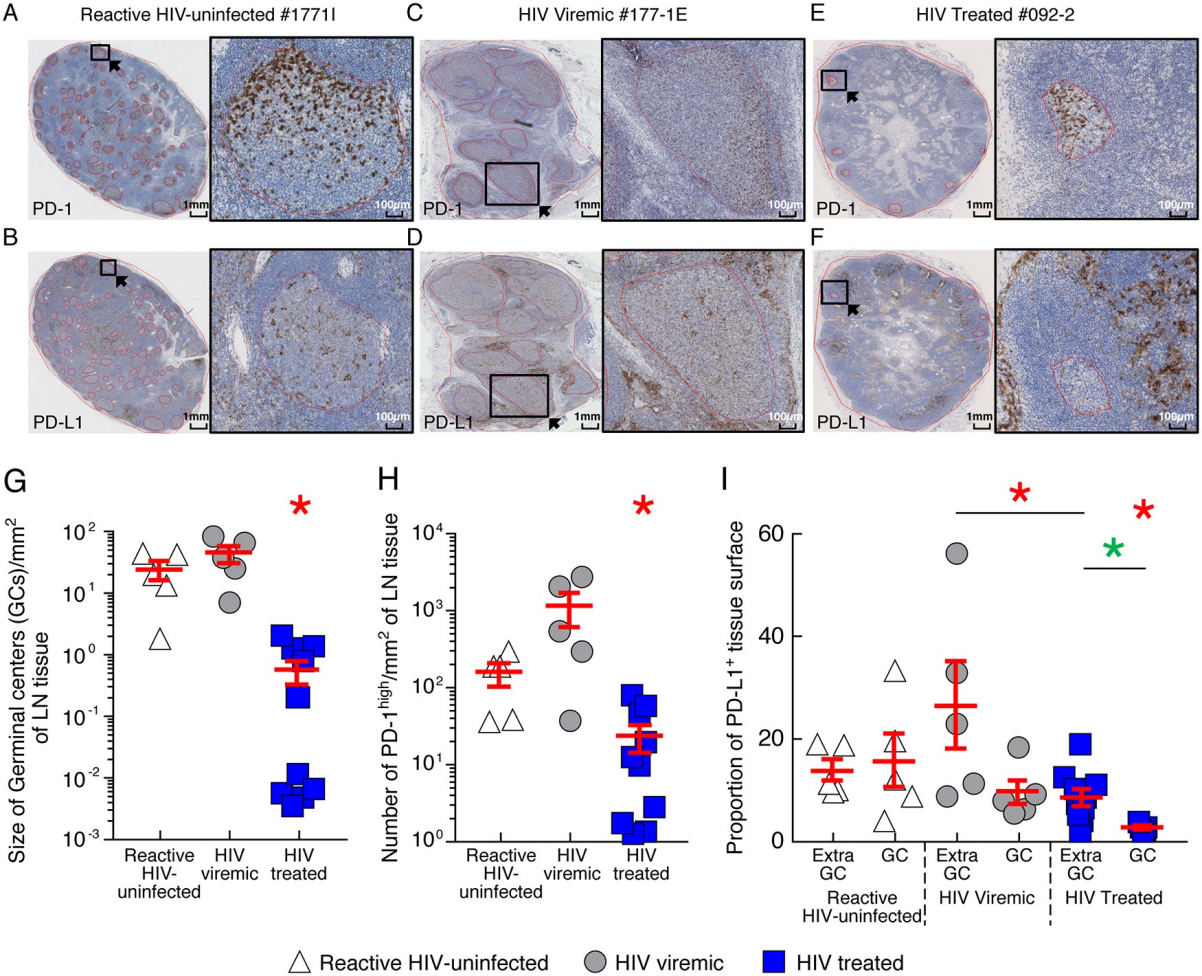
PD-1 and PD-L1 expression and tissue distribution in LN tissues. Representative examples of PD-1 (**A, C and E**) or PD-L1 (**B, D and F**) immunohistochemistry staining of LNs of reactive HIV-uninfected (#177-1I, **A-B**), viremic (#177-1E, **C-D**) and aviremic ART treated HIV-infected individual (#092–2, **E-F**). **(G)** Size of GCs per mm^2^ of LN tissue of reactive HIV-uninfected (N = 5), viremic (N = 5) and aviremic ART treated HIV-infected individual (N = 10). **(H)** Number of PD-1^high^ cells per mm^2^ of LN tissue of reactive HIV-uninfected (N = 5), viremic (N = 5) and aviremic ART treated HIV-infected individual (N = 10). **(I)** Proportion of PD-L1^+^ tissue surface of reactive HIV-uninfected (N = 5), viremic (N = 5) and aviremic ART treated HIV-infected individual (N = 10). Red circles represent GCs (**A-F**). Black squares correspond to the magnified areas (**A-F**). PD-1 expressing cells and PD-L1-positive areas were quantified using defined algorithms within total LN tissue area and within each GCs. White triangles correspond to reactive HIV-uninfected subjects, grey circles correspond to HIV-infected viremic individuals and blue squares correspond to aviremic ART treated HIV-infected individuals **(G-I)**. Red bars correspond to mean ± SEM **(G-I)**. Red stars indicate statistical significance (*P*<0.05) for inter-group comparisons and green stars indicate statistical significance (*P*<0.05) for inter-region comparisons. Statistical significance (*P* values) was obtained using one-way ANOVA (Kruskal-Wallis test) followed by Wilcoxon Matched-pairs two-tailed Signed Rank test (inter-group comparisons) or by Mann Whitney test (inter-region comparisons).

The representative examples and the cumulative data indicate that PD-1 and PD-L1 expressing cells were detected in both GC and extra-follicular areas of HIV-uninfected subjects, viremic and cART treated HIV-1 infected individuals (Fig 3A–3F). Comparison of serial immuno-labeled sections showed that cells expressing PD-1 were predominantly lymphocytes, while PD-L1 expressing cells were predominantly mononucleated histiocytes or dendritic cells. Cells expressing high levels of PD-1 (PD-1^high^ cells) were essentially detected in the GC areas. Consistent with previous study [24], the size of GCs/mm^2^ and the number of PD-1^high^ cells/mm^2^ of treated aviremic HIV-1 infected individuals were significantly reduced as compared to viremic untreated HIV-1 infected individuals (*P*<0.05) (Fig 3G and 3H).

Notably, due to the specific morphology of LN PD-L1-positive cells harboring cytoplasmic prolongations, we estimated the proportion of PD-L1 positive tissue surface as previously described [43]. Interestingly, the proportion of PD-L1 positive tissue surface was significantly lower in GCs than in extra-follicular areas of treated aviremic HIV-1 infected individuals (7.2% *versus* 2.2%; *P*<0.05) (Fig 3I). In addition, the proportion of PD-L1 positive tissue surface was significantly reduced in both GCs and extra-follicular areas of ART treated HIV-1 infected individuals as compared to viremic HIV-1 infected individuals (GC, 2.2% *versus* 9.4%; *P*<0.05; extra-follicular, 7.2% *versus* 26.4%; *P*<0.05) (Fig 3I).

Taken together, these results suggest that due to the different distribution in PD-L1 expression between extra-follicular and GC areas, the strength of the inhibitory signal resulting from PD-1/PD-L1 interactions may be weaker within GCs. The initiation of cART further influences the expression of both PD-1 and PD-L1 being associated with significant reduction in both extra-follicular and GC areas.

### PD-1/PD-L1 interaction modulates HIV-1 production in LN of treated aviremic HIV-infected individuals

**Fig 4:**
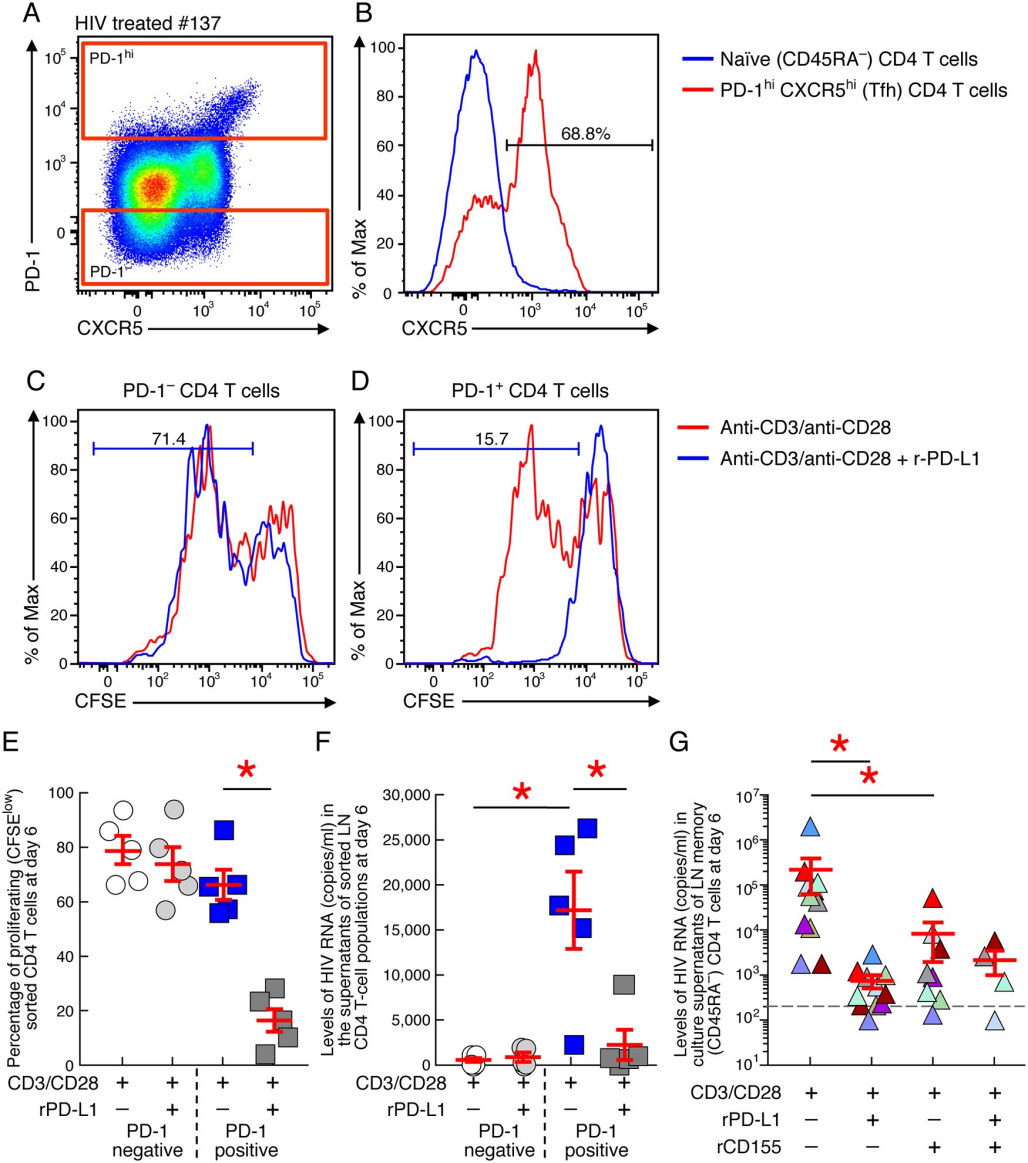
PD-1/PD-L1 interaction modulates HIV-1 transcription/production in LN of treated aviremic HIV-infected individuals. **(A)** Gating strategy used to sort LN PD-1^+^ and PD-1^-^ memory (CD45RA^-^) CD4 T cells from one representative aviremic ART treated HIV-infected individual (#137). **(B)** Representative flow cytometry profile of LN memory (CD45RA^-^) PD-1^+^ CD4 T cells expressing CXCR5 of one aviremic ART treated HIV-infected individual (#137). Representative flow cytometry profile of proliferating (CFSE low) LN PD-1^-^ **(C)** and PD-1^+^ **(D)** CD4 T cells following anti-CD3/anti-CD28 stimulation in the presence or in absence of recombinant PD-L1 protein of one aviremic ART treated HIV-infected individual (#137). **(E)** Cumulative data of the percentage of proliferating (CFSE low) LN PD-1^-^ and PD-1^+^ CD4 T cells following anti-CD3/anti-CD28 stimulation in the presence or in absence of recombinant PD-L1 protein of aviremic ART treated HIV-infected individuals (N = 5). **(F)** Cumulative data of the levels of HIV-1 RNA (copies/ml) in the culture supernatants at day 6 of LN PD-1^-^ and PD-1^+^ CD4 T cells following anti-CD3/anti-CD28 stimulation with emtricitabine in presence or in absence of recombinant PD-L1 protein of aviremic ART treated HIV-infected individuals (N = 5). **(G)** Cumulative data of the levels of HIV-1 RNA (copies/ml) in the culture supernatants at day 6 of sorted LN memory (CD45RA^-^) CD4 T cells following anti-CD3/anti-CD28 stimulation in the presence or in absence of recombinant CD155 and/or PD-L1 proteins of aviremic ART treated HIV-infected individuals (N = 10). Circles represent sorted LN PD-1^-^ CD4 T cells and squares represent sorted LN PD-1^+^ CD4 T cells **(E-F)**. Red bars correspond to mean ± SEM **(E-G)**. Red stars indicate statistical significance (*P*<0.05). Statistical significance (*P* values) was obtained using one-way ANOVA (Kruskal-Wallis test) followed by ratio Paired-t test (**E-G**).

We then investigated whether PD-1 was functionally active on LN PD-1^+^/Tfh cells. To address this issue, PD-1^high^ and PD-1-negative LN memory (CD45RA^-^) CD4 T cells were sorted from 5 treated aviremic HIV-infected individuals (Fig 4A). Consistent with a previous study, Tfh cells represented about 69% of the sorted PD-1^high^ memory CD4 T cells (Fig 4B) [24]. Sorted cell-populations were labelled with carboxyfluorescein (CFSE) and stimulated with coated anti-CD3/anti-CD28 monoclonal antibodies (MAbs) in the presence or in the absence of PD-L1 recombinant protein. The impact of PD-L1/PD-1 interaction on T-cell proliferation was assessed by flow cytometry based assay, while the reactivation of HIV-1 production was assessed using HIV-1 RNA detection in day 6 culture supernatants in the presence of emtricitabine to prevent *de novo* HIV-1 infection and virus replication. The cumulative data indicated that PD-L1 recombinant protein did not influence TCR-mediated T-cell proliferation and/or reactivation of HIV-1 production in LN PD-1-negative memory CD4 T cells (*P*>0.05) (Fig 4C, 4E and 4F). However, PD-L1 recombinant protein significantly reduced both TCR-mediated T-cell proliferation and reactivation of HIV-1 production in LN PD-1^+^/Tfh cells (*P*<0.05) (Fig 4D–4F). Similar to PD-L1 recombinant protein, CD155 recombinant protein significantly reduced TCR-mediated reactivation of HIV-1 production from LN memory (CD45RA^-^) CD4 T cells (*P*<0.05) (Fig 4G). However, the combination of PD-L1 and CD155 proteins did not further inhibit TCR-mediated reactivation of HIV-1 production as compared to either individual protein (*P*>0.05) (Fig 4G), suggesting that the negative signal arising upon PD-1 engagement was already sufficient to suppress TCR-mediated HIV production.

These results indicate that PD-1 and TIGIT are functionally active on LN PD-1^+^/Tfh cells and that the interaction with their ligands reduced TCR-mediated reactivation of HIV-1 production in LN PD-1^+^/Tfh cells of cART treated HIV-infected individuals *in vitro*.

### LN migratory (CD1c^high^CCR7^+^CD127^+^) dendritic cells expressing PD-L1 or PD-L2 modulate HIV-1 transcription/production in LN of treated aviremic HIV-infected individuals

**Fig 5:**
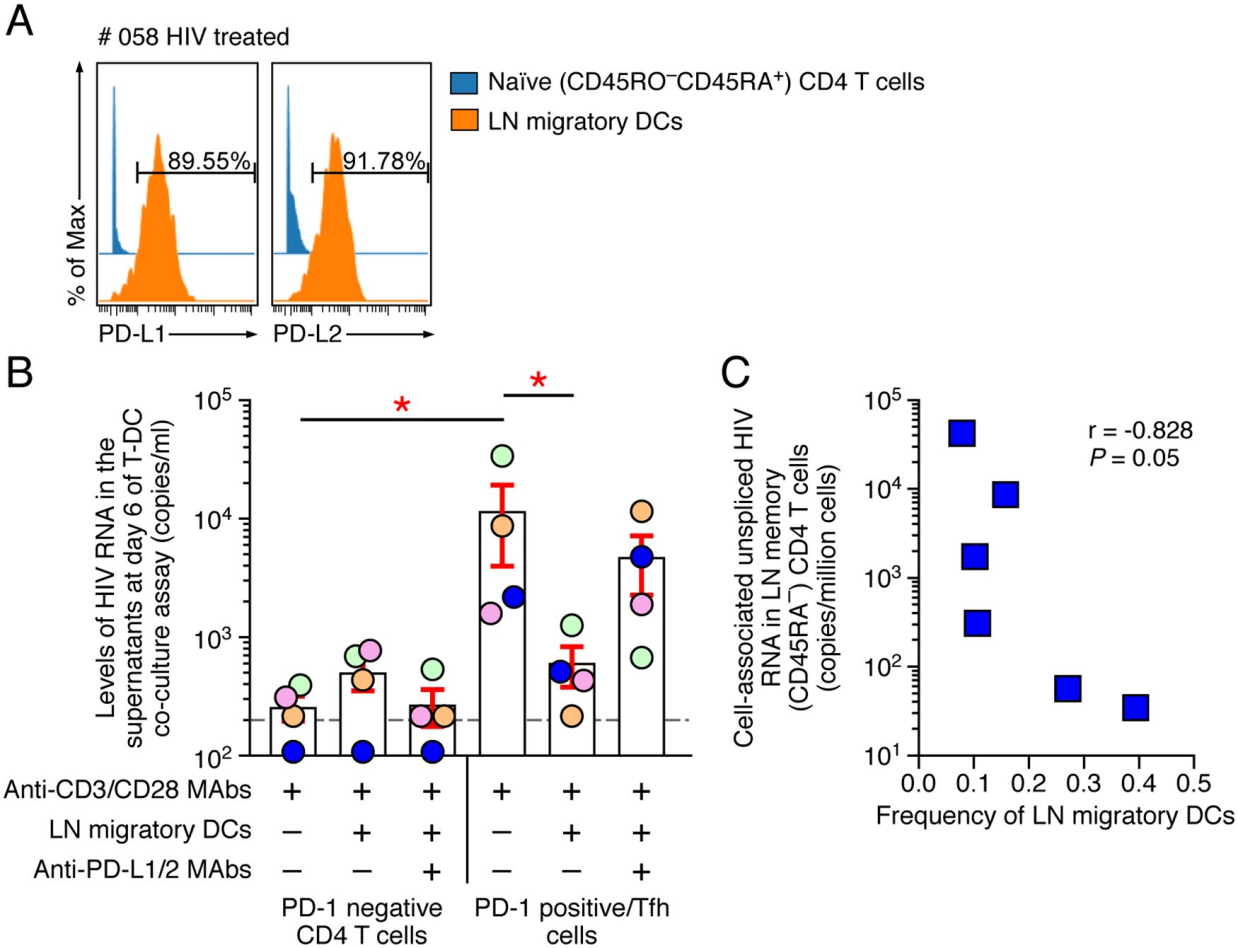
LN migratory dendritic cells expressing PD-L1 and/or PD-L2 modulate HIV-1 transcription/production in LN of treated aviremic HIV-infected individuals. **(A)** Level of expression of PD-Ls on LN (CD1c^high^CCR7^+^CD127^+^) migratory DCs from one representative aviremic ART treated HIV-infected individual (#058). **(B)** Cumulative data of the levels of HIV-1 RNA (copies/ml) in the culture supernatants at day 6 of LN PD-1^-^ or PD-1^+^ CD4 T cells following anti-CD3/anti-CD28 stimulation and co-culture with autologous LN migratory DCs in the presence or absence of blocking anti-PD-L1/L2 MAbs (N = 4). **(C)** Correlation between the levels of cell-associated unspliced HIV-1 RNA detected in LN memory (CD45RA^-^) CD4 T cells and the frequencies of LN migratory DCs in aviremic ART treated HIV-infected individuals (N = 6). HIV-infected individuals were color-coded **(B)**. Red stars indicate statistical significance (*P*<0.05). Statistical significance (*P* values) was obtained using one-way ANOVA (Kruskal-Wallis test) followed by ratio Paired-t test **(B)** or using Spearman rank test for correlations **(C)**.

We next explored the potential role of LN migratory DCs expressing PD-L1 and/or PD-L2 to modulate TCR-mediated reactivation of HIV-1 production from LN CD4 T cells expressing or not PD-1. To address this issue, PD-1-positive and PD-1-negative LN memory (CD45RA^-^) CD4 T cells were sorted from 4 treated aviremic cART treated individuals. Sorted cell-populations were stimulated with coated anti-CD3/anti-CD28 MAbs in the presence or in the absence of autologous LN migratory DCs cultured with or without blocking anti-PD-L1/2 MAbs. Reactivation of HIV-1 production was assessed using HIV-1 RNA detection in day 6 culture supernatants in the presence of emtricitabine. Of note, sorted LN migratory DCs expressed more than 80% of PD-L1 and/or PD-L2, respectively (Fig 5A).

As expected, cumulative data indicated that LN migratory DCs did not influence TCR-mediated reactivation of HIV-1 production in LN PD-1-negative memory CD4 T cells (*P*>0.05) (Fig 5B). However, LN migratory DCs expressing >80% PD-L1/PD-L2 significantly reduced TCR-mediated reactivation of HIV-1 production in LN PD-1^+^/Tfh cells (*P*<0.05) (Fig 5B). Notably, treatment of the cultures with anti-PD-L1/2 blocking MAbs partially restored the levels of HIV-1 RNA in the culture supernatants of LN PD-1^+^/Tfh cells (Fig 5B).

Interestingly, the frequencies of LN migratory DCs of cART treated HIV-infected individuals inversely correlated with the levels of cell-associated HIV-1 *gag* RNA found in sorted LN memory CD4 T cells (r = -0.828; *P* = 0.05) (Fig 5C).

Taken together, these data demonstrate that LN migratory DCs could modulate HIV-1 transcription in LN of treated aviremic HIV-infected individuals through a mechanism involving PD-L/PD-1 interactions.

### Anti-PD-1 monoclonal antibody Pembrolizumab reactivates HIV-1 replication from latently infected CD4 T cells *in vitro*

Additional evidence in support of the role of PD-1/PD-L1/2 interactions in regulating TCR-mediated HIV-1 reactivation was obtained evaluating the efficiency of anti-PD-1 MAb (pembrolizumab) to reactivate HIV-1 from latently infected resting memory CD4 T cells isolated from blood using a “modified” quantitative viral outgrowth assay (QVOA)[44]. For this purpose, resting memory CD4 T cells isolated from aviremic long-term treated HIV-1 infected individuals were cultured under different experimental conditions including 1) unstimulated (negative control), 2) stimulated with anti-CD3/anti-CD28 MAbs for 3 days in the presence of IL-2 (positive control), 3) exposed to isotype control, and 4) exposed to clinically relevant concentration of pembrolizumab for 14 days in the presence of autologous irradiated CD8-depleted PBMCs that include IC-L expressing cells. Of note, the use of autologous CD8-depleted PBMCs prevented the increase of transcriptional noise induced by mixed leukocyte reaction generated by the use of heterologous PBMCs and, the irradiation of CD8-depleted PBMCs prevented the reactivation of HIV-1 transcription from the feeder cells[44]. The QVOA was performed in the absence of ART to allow amplification of the reactivated virus and in a multiple replicate/limiting dilution format to allow the estimation of frequencies.

**Fig 6:**
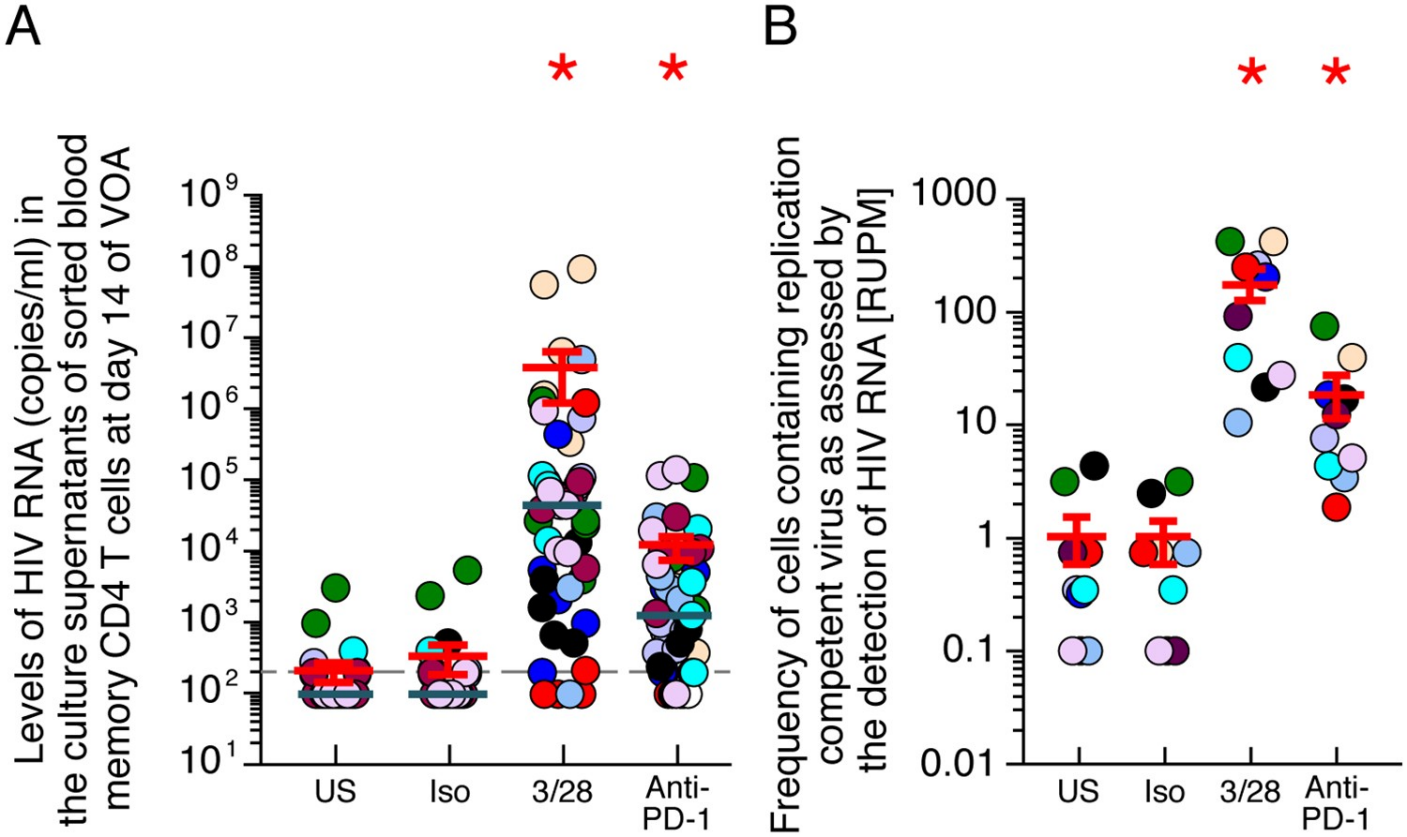
Anti-PD-1 monoclonal antibody Pembrolizumab reactivates HIV-1 replication from latently infected CD4 T cells *in vitro*. **(A)** Levels of HIV-1 RNA produced in the culture supernatants at day 14 of VOA of sorted blood resting memory CD4 T cells isolated from treated aviremic HIV-1-infected individuals (N = 10; 5 replicates per condition). Undetectable values were arbitrarily defined as 100 HIV-1 RNA copies/ml. **(B)** Estimated frequencies of inducible replication competent virus as measured by Replication Competent Unit per million (RUPM) in sorted blood resting memory CD4 T cells. Red bars correspond to mean ± SEM **(A-B)**. Green bars correspond to median **(A)**. HIV-infected individuals were color-coded **(A-B)**. Red stars indicate statistical significance (*P*<0.05) as compared to unstimulated condition **(A-B)**. Statistical significance (*P* values) was obtained using One-way ANOVA (Kruskal-Wallis test) followed by Wilcoxon Matched-pairs two-tailed Signed Rank test **(A)** or using Extreme Limiting Dilution analysis (ELDA) (http://bioinf.wehi.edu.au/software/elda/) **(B)**. “US” corresponds to unstimulated, “Iso” corresponds to isotype control, “3/28” corresponds to anti-CD3/anti-CD28 MAbs, “anti-PD-1” corresponds to Pembrolizumab.

The reactivation of HIV-1 replication induced in the various conditions was then assessed by HIV-1 RNA detection in day 14 culture supernatants using Roche Taqman assay as previously described and by the estimation of the frequencies of infected cells [44]. The levels of HIV-1 RNA induced under the various conditions were generated using the 5 replicates of the highest cell concentration (5.10^5^ cells/condition) of the “*modified* QVOA”. The cumulative data generated from 10 treated aviremic HIV-1 infected individuals indicated that pembrolizumab induced significantly higher levels of HIV-1 RNA in the culture supernatants as compared to untreated cultures or cells exposed to isotype control (*P*<0.05) (Fig 6A). The frequencies of cells containing replication competent virus were then evaluated in all conditions by the detection of HIV-1 RNA in QVOA supernatants and expressed as RNA-unit per million (RUPM) [45]. The results indicate that the average RUPM frequency following pembrolizumab exposure was significantly higher as compared to the unstimulated cells or cells exposed to the isotype control and represented about 14 cells containing replication competent virus per million (*P*<0.05) (Fig 6B).

Taken together, these data demonstrate that anti-PD-1 MAbs can efficiently reverse HIV-1 latency *in vitro*.

## Discussion

Increasing number of evidences indicate that B-cell follicles might be anatomical sanctuaries for active transcription in both HIV/SIV viremic controllers [22, 23, 26] and in ART treated aviremic HIV-infected individuals [24]. While multiple mechanisms may be involved in the regulation of HIV transcription, recent studies suggested that IC molecule expression may contribute to control HIV-1 transcription and therefore the maintenance of HIV latency in HIV-infected memory CD4 T cells [35–37]. Interestingly, we recently showed that Tfh cells expressing high levels of PD-1 serve as a major site of active and persistent virus transcription in ART treated aviremic individuals [24]. These observations prompted us to investigate the involvement of IC/IC-L interactions in the regulation of HIV-1 transcription in lymph node tissues.

We showed that Tfh cells, predominantly co-expressed PD-1 and TIGIT directly *ex vivo* in HIV-uninfected, viremic and aviremic ART treated HIV-infected individuals. We subsequently showed that PD-1 and TIGIT expressed on LN CD4 T-cell populations, including PD-1^+^/Tfh cells, were functionally active. Of note, in the present manuscript, we cannot exclude that LN PD-1^+^/Tfh cells may also encompass T follicular regulatory (TFR) cells.

Analysis of immunostained LN tissue sections indicated that PD-L1 expressing cells were detected in both extra-follicular and follicular regions of untreated viremic HIV-1 infected individuals and ART initiation was associated with a significant reduction of the proportion of PD-L1 positive tissue surface, even more pronounced in GC areas. An in-depth mass cytometry analysis revealed that PD-L1, PD-L2 and CD155 were predominantly co-expressed on a specific LN CD1c^high^ DC subpopulation expressing markers of migratory DCs *i*.*e*. CCR7 and CD127 [41, 42]. Consistent with previous studies, IC-Ls were also detected at low levels on blood monocytes and on blood and LN B-cell populations including GC B cells [38, 46, 47]. Interestingly, the frequency of migratory DCs directly correlated with HIV-1 viral load and was significantly reduced in aviremic ART treated HIV-infected individuals, thus indicating that HIV-1 replication influences the frequency of migratory DCs.

Migratory DCs differ from tissue to tissue but share the capacity to transport antigens to the draining LNs during both homeostatic conditions and infections [42]. In lymph nodes, migratory DCs preferentially locate at the T cell-B cell border where they contribute to induce Tfh-dependent antibody responses [48, 49].

Multiple cellular parameters such as TCR-mediated signaling, cytokine and chemokine stimulations or epigenetic DNA modifications can trigger reactivation of HIV-transcription [50], which might be in turn modulated by various mechanisms. In the present study, we investigated whether IC-Ls in general and migratory DCs expressing IC-Ls, in particular, could modulate HIV transcription in LN CD4 T cells including Tfh. Consistent with previous study, LN PD-1^+^/Tfh cells produced significantly higher levels of HIV-1 RNA than PD-1-negative CD4 T cells [24]. In particular, TCR-mediated HIV production of PD-1^+^/Tfh cells was strongly reduced *in vitro* in presence of recombinant PD-L1 or CD155.

More importantly, we demonstrated that LN migratory DCs could modulate TCR-induced HIV-1 transcription in LN by a mechanism involving PD-L/PD-1 interactions. Indeed, migratory DCs specifically regulated TCR-mediated HIV production of PD-1-positive CD4 T cells and anti-PD-L1/2 blocking MAb treatment partially restored the levels of HIV-1 RNA detected in PD-1-positive CD4 T-cell supernatants only. These data indicate that LN migratory DCs expressing IC-Ls may more efficiently restrict HIV-1 transcription in the extra-follicular areas *versus* GCs. In addition, the frequency of migratory DCs inversely correlated with HIV-1 transcription from LN memory CD4 T cells, suggesting that IC-L expressing migratory DCs might contribute to control HIV-1 transcription and maintain HIV-1 latency in extra-follicular areas. Notably, recent studies indicated that LN migratory DCs may harbor tolerogenic properties by a mechanism involving the production of immunosuppressive cytokines such as TGF-β and IL-10 and the induction of regulatory T cells [51–53].

Finally, we postulated that inhibition of PD-1/PD-Ls interactions might reverse HIV-1 latency and evaluated the efficiency of anti-PD-1 MAbs (pembrolizumab) to reactivate HIV-1 from latency using a “modified” QVOA. We demonstrated using primary CD4 T cells isolated from aviremic ART treated HIV-infected individuals and co-cultured with autologous irradiated CD8-depleted PBMCs, that clinically relevant concentration of pembrolizumab could efficiently reverse HIV-1 latency *in vitro*. While the precise mechanism regulating HIV latency in blood remains to be established, one may postulate that IC-L expressing monocytes may contribute to effects observed upon PD-1 blockade *in vitro*. These data support the recent observations suggesting the involvement of PD-1 in the establishment of HIV latency [36]. In addition, while one study found no effect [54], two other studies underscored the potential of PD-1 blockade in HIV cure strategies in combination or not with additional latency reversing agents [36, 55]. Therefore, immune checkpoint blocking antibodies (ICBs) represent a novel form of latency reversing agent that are currently being explored in the context of HIV functional cure [56]. Indeed, ICBs may potentially on one hand reverse HIV latency, thereby allowing for the expression of HIV proteins on the cell surface, and on the other hand, rescue the function of exhausted HIV-specific CD8 T cells to facilitate the elimination of reactivated cells [57]. In this regard, a phase II dose-escalation study of anti-PD-L1 antibody therapy in HIV-infected individuals was recently terminated due to safety concerns [58, 59]. Interestingly however, the data collected indicated an increase in Gag-specific CD4 and CD8 T-cell responses in a fraction of individuals with no changes in levels of cell associated HIV RNA or DNA in blood [58]. Unfortunately, no data were collected regarding the impact of ICB treatment on the tissue reservoirs. Taken together, these data indicate that future clinical trials based on ICBs should probably carefully estimate the risk-benefit ratio for HIV-infected individuals on stable suppressive ART and consider a thorough evaluation of the impact of ICB treatment on both blood and tissue reservoirs.

Recent studies suggested that IC signaling may contribute to maintain HIV-1 latency in HIV-1 infected memory CD4 T cells [36, 37, 55], however, these studies were performed exclusively on cells isolated from blood, while the present study provides four lines of evidence on the role of IC/IC-L interactions in regulating HIV transcription in LN tissues that include: 1) PD-1/PD-L1 interactions strongly impact the reactivation of TCR-mediated HIV-1 production from LN memory CD4 T cells 2) the modulation of HIV-1 transcription by LN migratory DCs through a mechanism involving PD-L/PD-1 interactions 3) the relationship between the levels of HIV-1 transcription and the frequency of PD-L1/2 expressing migratory DCs and 4) PD-1 blockade with anti-PD-1 monoclonal antibody treatment can reactivate of HIV-1 replication from latently infected CD4 T cells.

These findings represent a step forward in our understanding of the mechanism regulating the persistence of HIV transcription in lymphoid tissues.

## Materials and methods

### Ethics statement

Fifty-seven HIV-1 infected adult volunteers and twelve HIV-uninfected subjects were enrolled in the present study. No statistical method was used to predetermine sample size. The present study was approved by the Institutional Review Board of the Centre Hospitalier Universitaire Vaudois, and all subjects which were adults gave written informed consent. The 57 HIV-1-infected individuals studied had a documented diagnosis of HIV-1 infection between 0.4 and 28.4 years. Treated HIV-1 infected individuals received ART treatment for 0.3 to 23.1 years. No exclusion criteria was implemented except with regards to the reactivation of HIV-1 latency from blood resting memory CD4 T-cell experiments, which were exclusively performed on cells isolated from treated aviremic HIV-1 infected individuals with undetectable viremia (HIV-1 RNA levels <50 copies per ml of plasma) for at least 12 months. Inguinal lymph node biopsies and blood samples from HIV-infected individuals were collected the same day. In addition, LN sections collected from HIV-uninfected individuals suffering from lymphadenopathy were also collected and were referred to as “reactive” LNs.

### Cell isolation

Blood mononuclear cells were isolated as previously described [24] and lymph node mononuclear cells were isolated by mechanical disruption as previously described [60]. Blood mononuclear cells and lymph node mononuclear cells were cryopreserved in liquid nitrogen for long-term storage.

### Cell culture

Cells were cultured in RPMI (Gibco; Life Technologies) containing 10% heat-inactivated FBS (Institut de Biotechnologies Jacques Boy), 100 IU/ml penicillin and 100 μg/ml streptomycin (Bio Concept).

### Antibodies

The following antibodies were used for sorting experiments: APC-H7-conjugated anti-CD3 (clone SK7), APC or FITC-conjugated anti-CD4 (clone RPA-T4), ECD-conjugated anti-CD45RA (clone 2H4), V450-conjugated anti-HLA-DR (clone G46-6), PE-Cy7-conjugated anti-CD25 (clone M-A251), PerCP-Cy5.5-conjugated anti-CD69 (clone L78), PE-Cy7-conjugated anti-PD-1 (clone EH12.1) and PE-conjugated anti-CXCR5 (clone MU5UBEE), APC-conjugated CD1c (L161) and PB-conjugated CD127 (HIL-7R-M21). All antibodies including purified coating anti-CD3 (clone UCHT1) and anti-CD28 (clone CD28.2) mAbs were purchased from BD (Becton Dickinson; CA, USA); and ECD-conjugated anti-CD45RA (clone 2H4) from Beckman Coulter (CA, USA) and APC-conjugated CD1c (L161) from Biolegend. Blocking anti-PD-L1 (MIH1) and anti-PD-L2 (MIH18) MAbs were purchased from eBioscience. The following antibodies were used for mass cytometry experiments: 113In-conjugated anti-CD8 (RPA-T8), 115In-conjugated anti-CD4 (RPA-T4), 139La-conjugated anti-CD3 (UCHT1), 141Pr-conjugated anti-CD45 (HI30), 142Nd-conjugated anti-CD19 (HIB19), 143Nd- conjugated anti-ICOS (C398.4A), 145Nd-conjugated anti-CD57 (HCD57), 146Nd-conjugated anti-IgD (IA6-2), 147Sm-conjugated anti-CD7 (CD7-6B7), 148Sm-conjugated anti-PD-L1 (29E.2A3), 149Sm-conjugated anti-CD127 (A019D5), 150Nd-conjugated anti-Lag-3 (11C3C65), 151Eu-conjugated anti-CD123 (6H6), 152Sm-conjugated anti-CD21 (BL13), 153Eu-conjugated anti-Tim-3 (F38-2E2), 154Sm-conjugated anti-TIGIT (MBSA43), 155Gd-conjugated anti-CD27 (L128), 156Gd-conjugated anti-CD10 (HI10α), 158Gd-conjugated anti-CD169 (7–239), 159Tb-conjugated anti-CCR7 (G043H7), 160Gd-conjugated anti-CD14 (M5E2), 161Dy-conjugated anti-CD1c (L161), 162Dy-conjugated anti-CD11c (Bu15), 163Dy-conjugated anti-CXCR3 (G025H7), 164Dy-conjugated anti-CXCR5 (51505), 165Ho-conjugated anti-CD45RO (UCLH1), 166Er-conjugated anti-CD155 (SKII.4), 167Er-conjugated anti-CD38 (HIT2), 168Yb-conjugated anti-CD66b (CD66α-B1.1), 169Yb-conjugated anti-CD45RA (HI100), 170Er-conjugated anti-CTLA-4 (14D3), 171Yb-conjugated anti-CD20 (clone), 172Yb-conjugated anti-PD-L2 (24F.10C12), 173Yb-conjugated anti-CXCR4 (12G5), 174Yb-conjugated anti-HLA-DR (L243), 175Lu-conjugated anti-PD-1(EH12.2H7) and 191Ir was used to label DNA. Antibodies against CD8, CD4, CD3, CD28, CD155 were purchased from Biolegend. Anti-CD57 was purchased from BD. All other antibodies were purchased from Fluidigm/DVS.

### Sorting of blood and LN CD4 T–cell populations

Cryopreserved blood mononuclear cells were thawed, enriched using EasySep Human CD4 T Cell Enrichment kit (StemCell Technologies, USA), stained with Aqua LIVE/DEAD stain kit (4 °C; 15 min) and then with anti-CD3 APC-H7, anti-CD4 FITC, anti-CD45RA ECD, anti-HLA-DR V450, anti-CD25 PE-Cy7 and anti-CD69 PerCP-Cy5.5 MAbs. Cryopreserved lymph node mononuclear cells were thawed and then stained with Aqua LIVE/DEAD stain kit (4°C; 15 min) and then with anti-CD3 APC-H7, anti-CD4 APC, anti-CD45RA ECD, anti-PD-1 PE-Cy7 and anti-CXCR5 PE (4°C; 25 min). Viable blood resting memory (CD45RA^-^HLA-DR^-^CD25^-^CD69^-^) CD4 T cells and viable LN memory (CD45RA^-^) CD4 T cells, PD-1^high^ and PD-1^-^ CD4 T-cell populations were sorted using FACSAria (Beckton & Dickinson). In all sorting experiments the grade of purity of the sorted cell populations was >98%.

### Mass cytometry

Freshly isolated matched blood and lymph node mononuclear cells were resuspended (10^6^ cells/ml) in complete RPMI medium and incubated (30 min; 4 °C) directly *ex vivo* (no permeabilization) with metal-conjugated antibodies directed against a panel of 37 parameters (Fluidigm/DVS Science) including lineage markers for T-cell, B-cell and antigen presenting-cell populations and ICs such as PD-1, CTLA-4, TIM-3, LAG-3, TIGIT as well as IC-ligands such as PD-L1, PD-L2 and CD155. Cells were washed and fixed (10 min; room temperature) with 2.4% PFA. Total cells were identified by DNA intercalation (1μM Cell-ID Intercalator, Fluidigm/DVS Science) in 2% PFA at 4 °C overnight. Labeled samples were acquired on a CyTOF1 instrument that was upgraded to CyTOF2 (Fluidigm) using a flow rate of 0.045 ml/min. Data were analyzed using Fluidigm Cytobank software package (Cytobank, Mountain View, CA). At least 100,000 events were acquired for each sample.

### CD4 T-cell proliferation

Sorted LN PD-1^high^ and PD-1^-^ memory (CD45RA^-^) CD4 T-cell populations (2 × 10^5^ cells) were washed twice, labeled with CFSE at 37°C for 7 min (Life Technologies) and cultured for 6 days at 37°C and 5% CO_2_ in 96-well U-bottom plates coated with 10 μg/ml anti-CD3 (BD) and 10 μg/ml anti-CD28 (BD) in presence or not of recombinant IC ligands at 100 μg/mL in complete RPMI. The proliferation of CD4 T-cell populations was assessed by quantifying the percentage of CFSE low cells on a LSR SORP cell analyzer (BD). Of note, all experiments were performed in presence of 75 nM emtricitabine to prevent re-infection of stimulated CD4 T cells.

### Histopathology

Lymph nodes were cut into slices and fixed in B-plus or formalin before routine processing and embedding in paraffin blocks. Serial tissue sections (4-μm) were stained according to standard routine protocols by using a Ventana benchmark platform (Roche) with antibodies against PD1 (192106 R&D Systems) and PD-L1 (sp263, Ventana). PD1 and PD-L1-imunostained slides were digitalized using a Hamamatsu Nanozoomer 1.0 scanner (model C9600-01) at 40× with the NDPScan software (v. 2.5.89). Scanning area and focus points were set manually. Image analysis was performed with the Tissue IA–specific module of the Slidepath Software Digital Image Hub (DIH) (version 4.0.7). The surfaces of the entire tissue section and of individual germinal centers (manually circumscribed) were measured. Cell density was estimated using the “measure stained cells algorithm” at 20× to quantify PD-1-positive cells, while PD-L1-positive areas were quantified using “measure stained area algorithm” at 20×.

### HIV-1 production

Sorted LN PD-1^high^ and PD-1^-^ memory (CD45RA^-^) CD4 T-cell populations (2 × 10^5^ cells) were cultured for 6 days at 37°C and 5% CO_2_ in 96-well U-bottom plates coated with anti-CD3 (BD) and anti-CD28 (BD) (10 μg/ml) in presence or in absence of recombinant IC ligands at 100 μg/mL in complete RPMI. In some experiments, the combination of two IC-ligands was also tested. Of note, all experiments were performed in presence of 75 nM emtricitabine to prevent re-infection of stimulated CD4 T cells. Supernatants were collected at days 6 and quantification of HIV-1 production was performed by assessing HIV-1 RNA levels by COBAS AmpliPrep/TaqMan HIV-1 Test (Roche; Switzerland) as previously described [44].

### T cell/DC co-culture assay

Freshly isolated LNMCs were stained with Aqua LIVE/DEAD stain kit (4°C; 15 min) and then with anti-CD1c APC, anti-CD127 PB, anti-CD3-PE, anti-CD4 APC-H7, anti-CD45RA ECD and anti-PD-1 PE-Cy7 (4°C; 25 min). Viable LN memory (CD45RA^-^) PD-1^+^ and PD-1^-^ CD4 T-cell populations and CD1c^high^CD127^high^ DCs were sorted using FACSAria (Beckton & Dickinson). In all sorting experiments the grade of purity of the sorted cell populations was >98%. Sorted CD4 T cell populations (10^5^ cells) were stimulated with anti-CD3/anti-CD28 MAbs (10μg/ml) or co-cultured with autologous DCs (ratio DCs/T 1∶10) in the presence or absence of blocking anti-PD-L1/2 MAbs (10 μg/ml) in 96-well U-bottom plates. All cultures were carried out in the presence of emtricitabine. Supernatants were collected at day 6 and quantification of HIV-1 production was performed by assessing HIV-1 RNA levels by COBAS AmpliPrep/TaqMan HIV-1 Test (Roche; Switzerland) as previously described [44]. PD-L1, PD-L2 and CD155 expression was assessed on sorted migratory DCs using mass cytometry.

### Quantification of Cell-associated RNA

Cell-associated HIV-1 RNA (CA-RNA) was assessed as previously described [24]. Briefly, CA-RNA was extracted from sorted CD4 T-cell populations (5 x 10^4^ cells) and subjected to DNase treatment (RNAqueous-4PCR Kit Ambion). RNA standard curves were generated after isolation and quantification of viral RNA from supernatant of ACH2 culture as previously described [61]. One step cDNA synthesis and pre-amplification were performed as previously described [62] using the following primers ULF1: 5'- ATG CCA CGT AAG CGA AAC TCT GGG TCT CTC TDG TTA GAC-3' UR1: 5’- CCA TCT CTC TCC TTC TAG C -3'. Real-time PCR was performed using Roche light Cycler 480II using the following primers: LambdaT: 5'-ATG CCA CGT AAG CGA AAC T -3'; UR2: 5'- CTG AGG GAT CTC TAG TTA CC-3' and probes: 56-FAM 5’-CAC TCA AGG/ ZEN/CAA GCT TTA TTG AGG C-3’ IABkFQ 35.

### Viral outgrowth assay (VOA)

Different cell concentrations (fivefold limiting dilutions, *i*.*e*., 5× 10^5^, 1 × 10^5^, 2 × 10^4^ and 4 × 10^3^ cells of sorted viable blood resting memory (CD45RA^-^HLA-DR^-^CD25^-^CD69^-^) CD4 T cells from aviremic ART treated HIV-infected individuals were co-cultured with autologous irradiated CD8-depleted blood mononuclear cells (10^6^ cells/ml), as previously described [44], in presence or in absence of blocking anti-PD-1 Mab, Pembrolizumab, at 5 μg/mL or isotype control (Eureka Therapeutics). As a positive control, sorted cells were stimulated for 3 days with anti-CD3 and anti-CD28 mAb-coated plates (10 μg/ml) in presence of IL-2 (50 units/ml). Supernatants were collected at days 0, 5 and 14. Medium was replaced at day 5. Quantification of replication competent HIV-1 was performed at 14 by assessing HIV-1 RNA levels by COBAS AmpliPrep/TaqMan HIV-1 Test (Roche; Switzerland) as previously described [44]. Wells with detectable HIV-1 RNA (≥200 HIV-1 RNA copies/ml) were referred to as HIV-1 RNA-positive wells. The frequency of latently HIV-1 infected CD4 T cells reactivated by IC MAbs was estimated by conventional limiting dilution methods using Extreme Limiting Dilution analysis (http://bioinf.wehi.edu.au/software/elda/) [63] and expressed in RNA-unit per million (RUPM) as previously described [44].

### Statistical analyses

Statistical significance (*P* values) was obtained using one-way ANOVA (Kruskal-Wallis test) followed by Mann-Whitney test or Wilcoxon Matched-pairs two-tailed Signed Rank test or ratio Paired-t test or Spearman rank test was used for correlations. Finally, Statistical significance (*P* values) was obtained using Extreme Limiting Dilution analysis (http://bioinf.wehi.edu.au/software/elda/) for comparison of HIV-infected cell frequencies. The analyses of multiple comparisons were taken into account for the calculation of statistical significance.

## Supporting information

S1 Fig IC molecule expression on blood and LN memory CD4 T-cell populations. Cumulative percentage of LAG-3, TIM-3 and CTLA-4 expression on blood (**A**) and LN (**B**) memory (CD45RA-) CD4 T-cell populations identified on the basis of PD-1 and/or CXCR5 expression of HIV-uninfected (N = 7), viremic (N = 10) and aviremic ART treated HIVinfected individuals (N = 10). White symbols correspond to HIV-uninfected individuals, grey symbols corresponds to HIV-1 viremic individuals and blue symbols correspond to HIVinfected aviremic ART treated individuals (**A-B**). Blood CD4 T-cell populations are represented as circles (**A**) whereas LN CD4 T-cell populations are represented as triangles (**B**). Red bars correspond to mean ± SEM (**A-B**). Red stars indicate statistical significance (* = *P*<0.05) for intra-group comparisons whereas green stars indicate statistical significance (* = *P*<0.05) for inter-population comparisons (**A-B**). Statistical significance (*P* values) was obtained using one-way ANOVA (Kruskal-Wallis test) followed by Mann Whitney test (intragroup comparisons) or Wilcoxon Matched-pairs two-tailed Signed Rank test (interpopulation comparisons).
(PDF)

S2 Fig Gating strategy for blood and LN mononuclear cell populations. Representative example of gating strategy for blood (**A**) monocytes (CD14^+^), B cell (CD19^+^) subpopulations and DC subsets of an aviremic ART treated HIV-infected individual and LN (**B**) B cell (CD19^+^) subpopulations and DC subsets of an aviremic ART treated HIVinfected individual.
(PDF)

S3 Fig IC-Ligand expression on blood or LN cell populations. Level of expression of PD-L1, PD-L2 or CD155 on various mononuclear cell populations from matched blood (**C**) and LN (**D**) of HIV-uninfected (#097), viremic (#124) and aviremic ART treated HIV-infected individual (#137).
(PDF)

S4 Fig Correlations between the frequency of IC-L expressing blood monocytes and HIV viral load and with duration of ART. Correlation between the levels of HIV viral load and the frequencies of PD-L1^+^ (**A**), PD-L2^+^ (**B**) and CD155^+^ (**C**) blood monocytes in viremic HIV-infected patients (N = 10) and between the frequencies of PD-L1^+^ (**D**), PD-L2^+^ (**E**) blood monocytes and duration of antiretroviral therapy (years) in treated HIV-infected patients (N = 10). Grey symbols correspond to HIV-1 viremic individuals (**A-C**) and blue symbols correspond to HIV-infected aviremic ART treated individuals (**D-E**). Statistical significance **(***P* values) was obtained using Spearman rank test for correlations.
(PDF)

S5 Fig IC-L expression on distinct DC sub-populations. Cumulative data of proportion of PD-L1^+^ (**A)**, PD-L2^+^ (**B**) and CD155^+^ (**C**) DCs among LN HLA-DR^+^CD1c^high^CCR7^+^CD127^+^ (referred to as “DP”) and LN HLADR^+^CD1c^high^CCR7^-^CD127^-^ (referred to as “DN”) DCs of HIV-uninfected (N = 7), viremic (N = 10) and aviremic ART treated HIV-infected individuals (N = 10). HIV-uninfected individuals are represented in circles, HIV viremics in triangles and HIV-infected ART treated individuals are represented in squares. “DP” and “DN” are color-coded. Red bars correspond to mean ± SEM (**A-C**). Red stars indicate statistical significance (* = *P*<0.05) (**A-C**). Statistical significance (*P* values) was obtained using one-way ANOVA (Kruskal-Wallis test) followed by Wilcoxon Matched-pairs two-tailed Signed Rank test.
(PDF)

S6 Fig Correlation between frequency of LN migratory DCs and Tfh cells and between IC-L expressing DCs with HIV viral load. Correlation between percentage of Tfh cells and frequencies of LN migratory DCs (**A**) and between mean signal intensity (MFI) of PD-1 on Tfh cells and mean signal intensity (MFI) of PD-L1 on LN migratory DCs (**B**) in untreated viremic HIV-infected individuals (N = 10). (**C**) Correlation between the levels of HIV viral load and the frequencies of LN PD-L1^+^ migratory DCs in viremic HIV-infected individuals (N = 10). (**D**) Correlation between the levels of HIV viral load and the frequencies of LN PD-L2^+^ migratory DCs in viremic HIVinfected individuals (N = 10). Grey symbols correspond to HIV-1 viremic individuals. Statistical significance (*P* values) was obtained using Spearman rank test for correlations.
(PDF)
