# Supplementary figures and images for: Lymph node migratory dendritic cells modulate HIV-1 transcription through PD-1 engagement
Source: PLoS Pathog. 2019 Jul 22;15(7):e1007918. doi: 10.1371/journal.ppat.1007918 (PMC6675123; doi:10.1371/journal.ppat.1007918)

Supplemental Figure 1

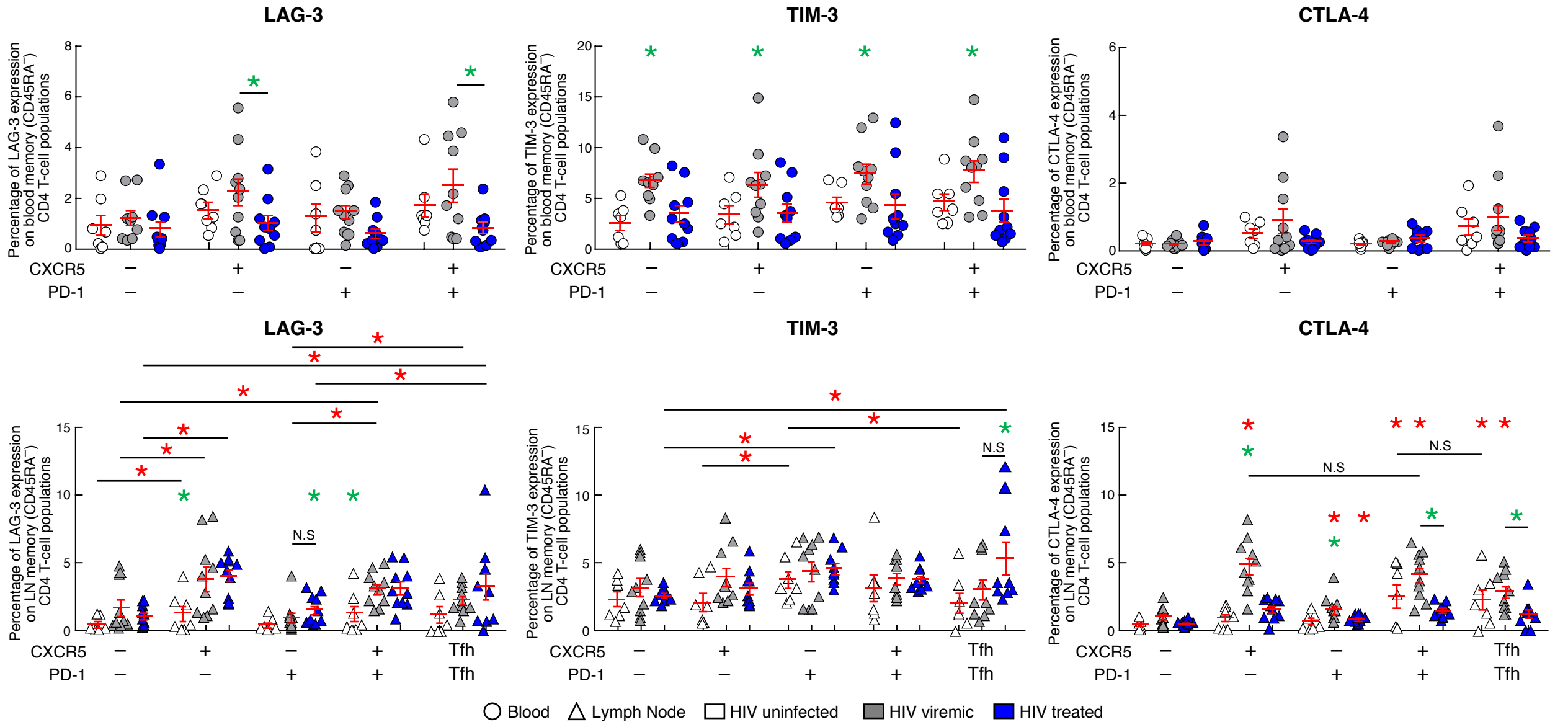

Supplement: S1 Fig — Cumulative percentage of LAG-3, TIM-3 and CTLA-4 expression on blood (A) and LN (B) memory (CD45RA-) CD4 T-cell populations identified on the basis of PD-1 and/or CXCR5 expression of HIV-uninfected (N = 7), viremic (N = 10) and aviremic ART treated HIVinfected individuals (N = 10). White symbols correspond to HIV-uninfected individuals, grey symbols corresponds to HIV-1 viremic individuals and blue symbols correspond to HIVinfected aviremic ART treated individuals (A-B). Blood CD4 T-cell populations are represented as circles (A) whereas LN CD4 T-cell populations are represented as triangles (B). Red bars correspond to mean ± SEM (A-B). Red stars indicate statistical significance (* = P<0.05) for intra-group comparisons whereas green stars indicate statistical significance (* = P<0.05) for inter-population comparisons (A-B). Statistical significance (P values) was obtained using one-way ANOVA (Kruskal-Wallis test) followed by Mann Whitney test (intragroup comparisons) or Wilcoxon Matched-pairs two-tailed Signed Rank test (interpopulation comparisons). (PDF) [file ppat.1007918.s001.pdf]

# Supplemental Figure 2

## Blood

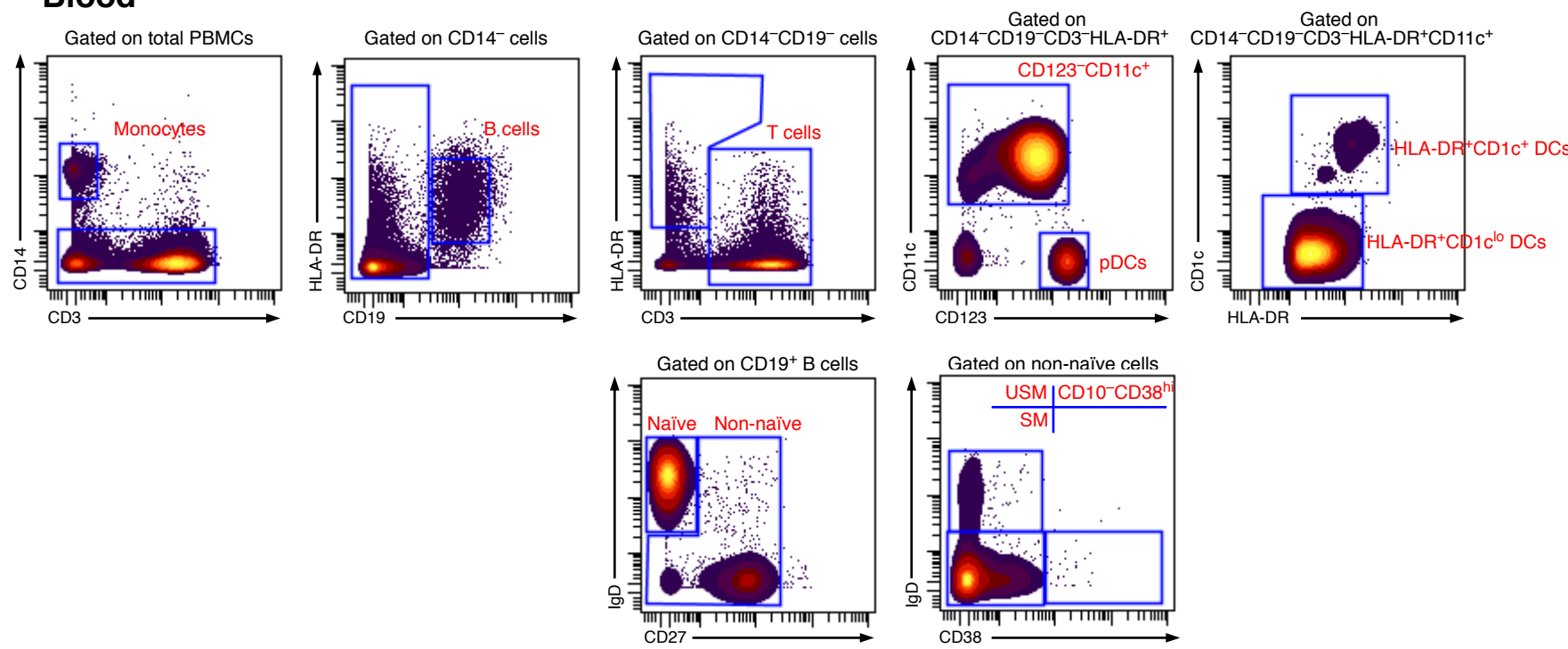

## LN

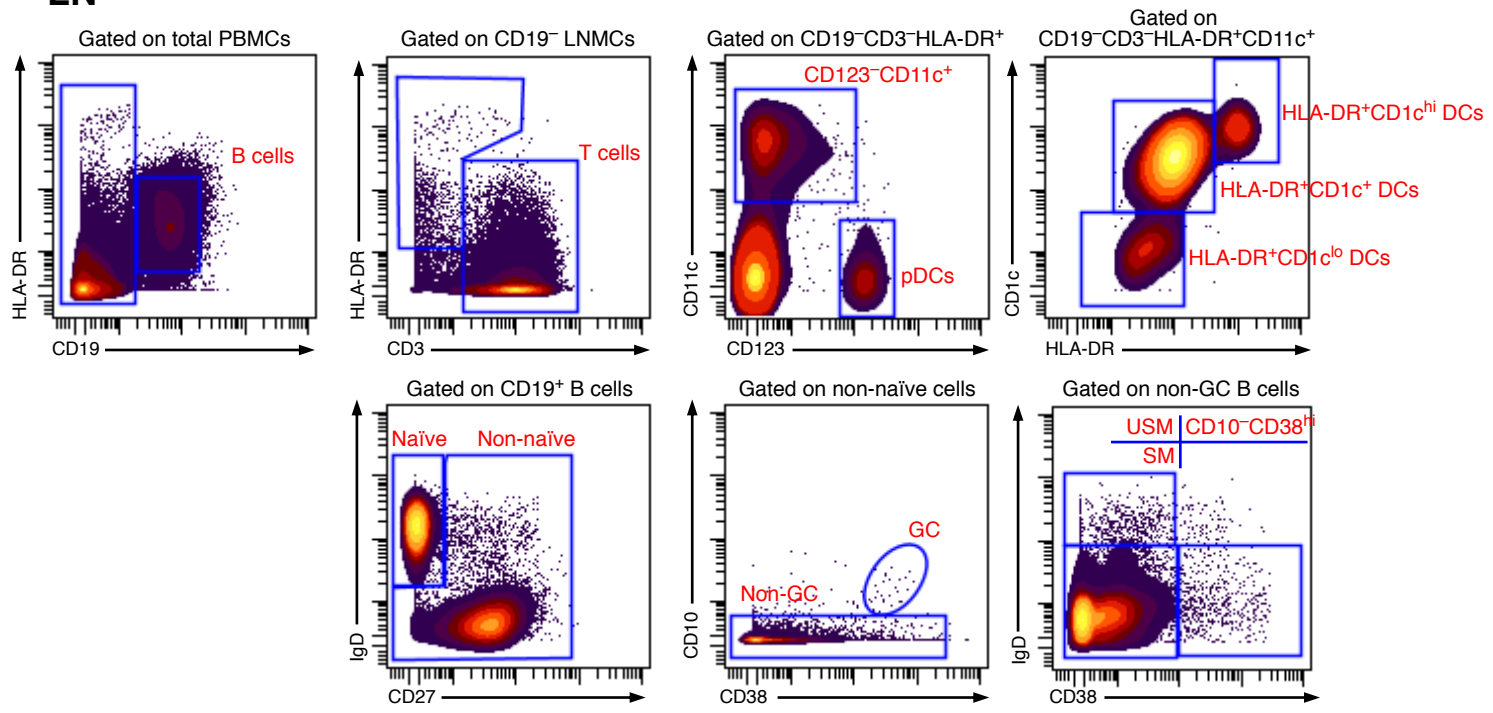

Supplement: S2 Fig — Representative example of gating strategy for blood (A) monocytes (CD14+), B cell (CD19+) subpopulations and DC subsets of an aviremic ART treated HIV-infected individual and LN (B) B cell (CD19+) subpopulations and DC subsets of an aviremic ART treated HIVinfected individual. (PDF) [file ppat.1007918.s002.pdf]

# Supplemental Figure 3

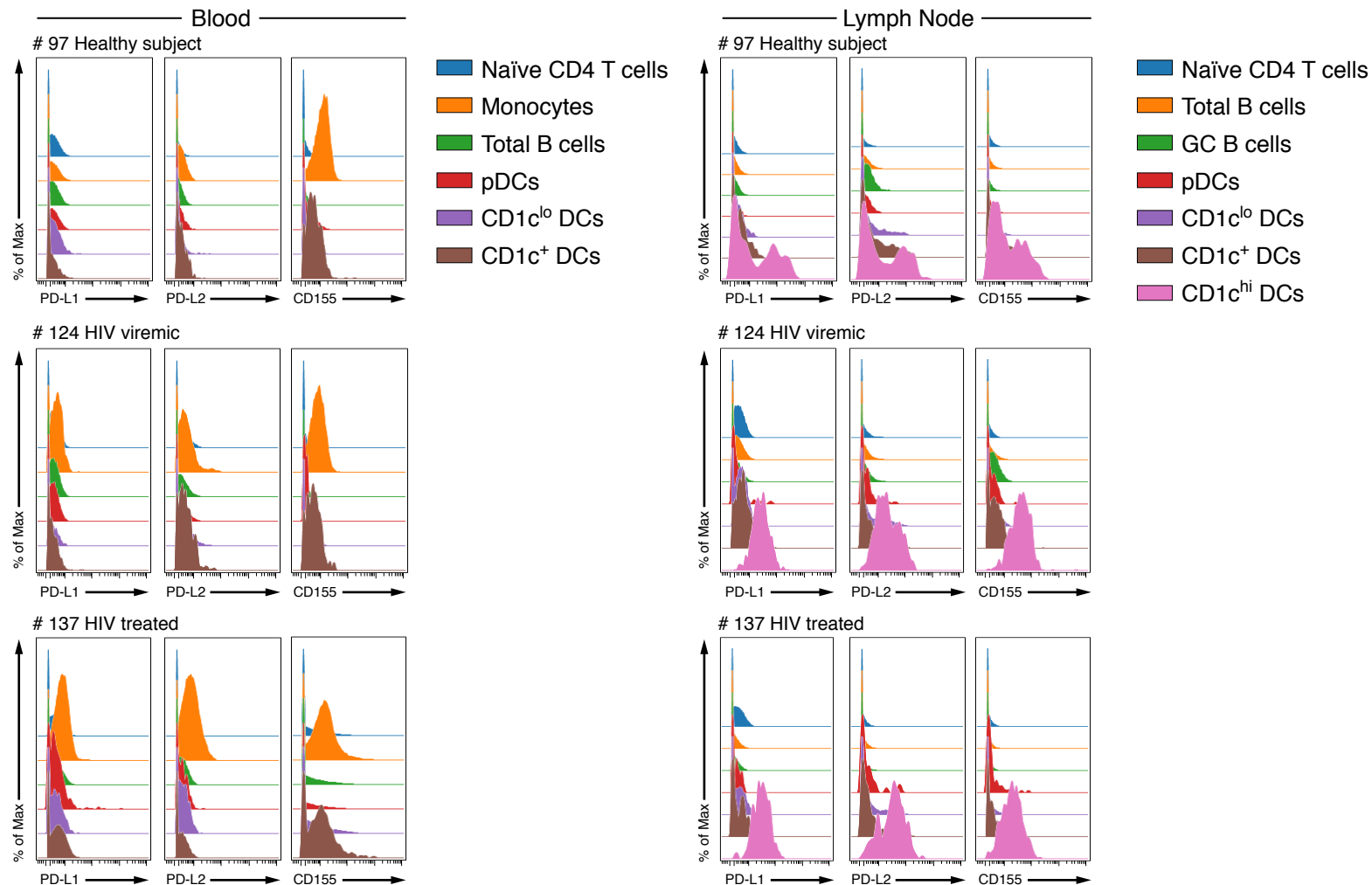

# Supplemental Figure 6

A

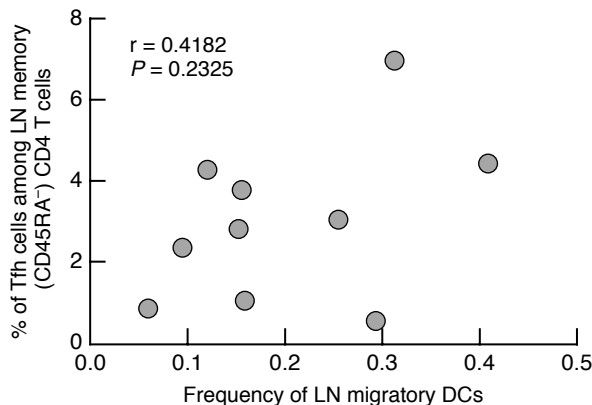

B

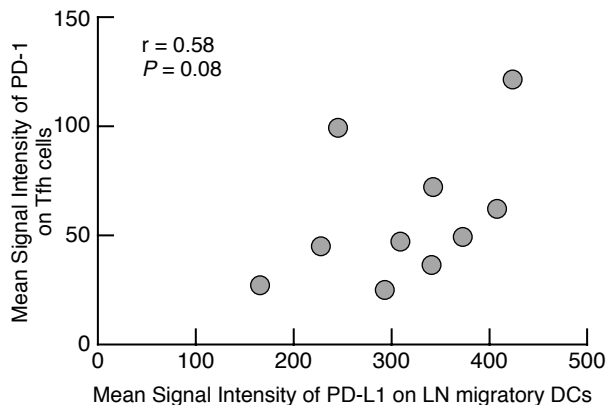

C

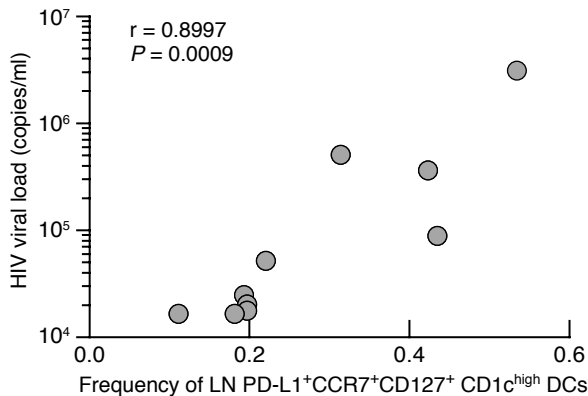

D

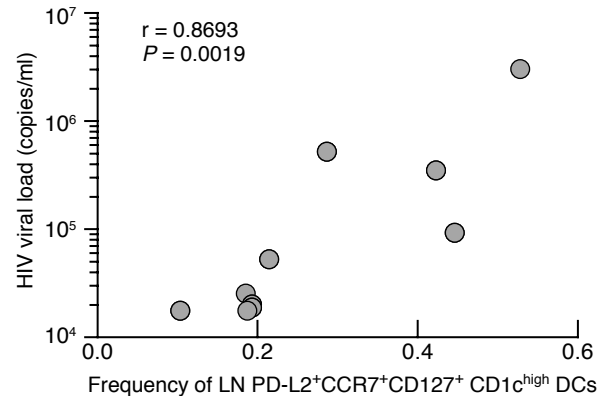

Supplement: S3 Fig — Level of expression of PD-L1, PD-L2 or CD155 on various mononuclear cell populations from matched blood (C) and LN (D) of HIV-uninfected (#097), viremic (#124) and aviremic ART treated HIV-infected individual (#137). (PDF) [file ppat.1007918.s003.pdf]

# Supplemental Figure 4

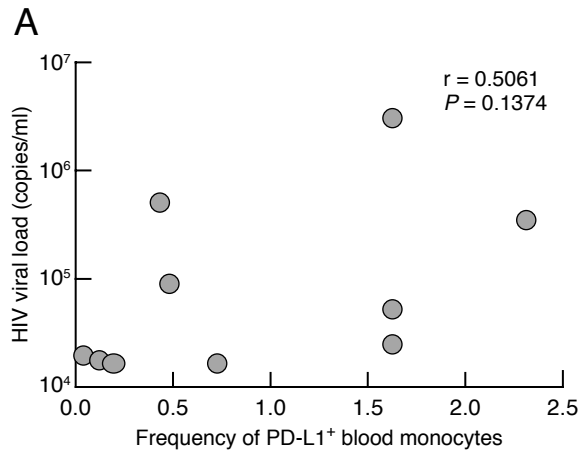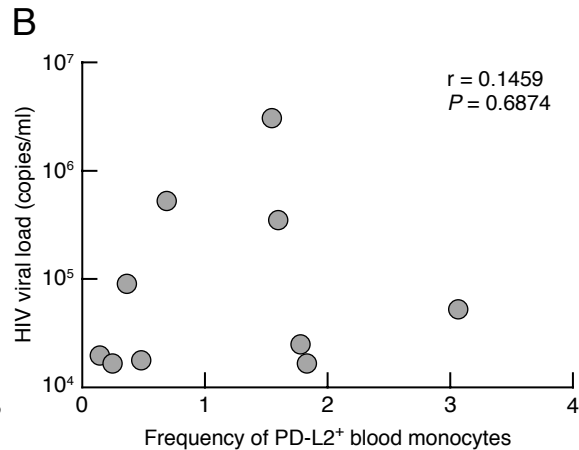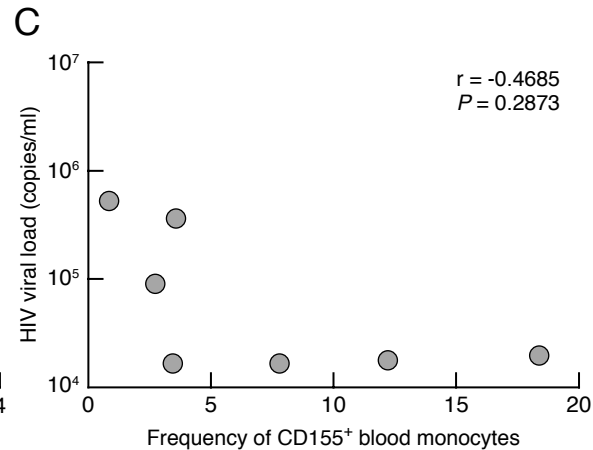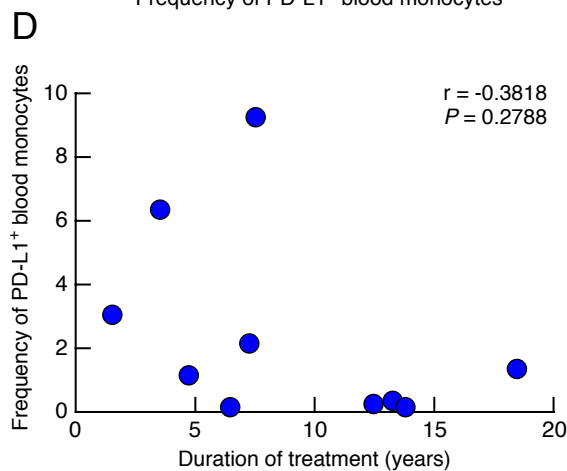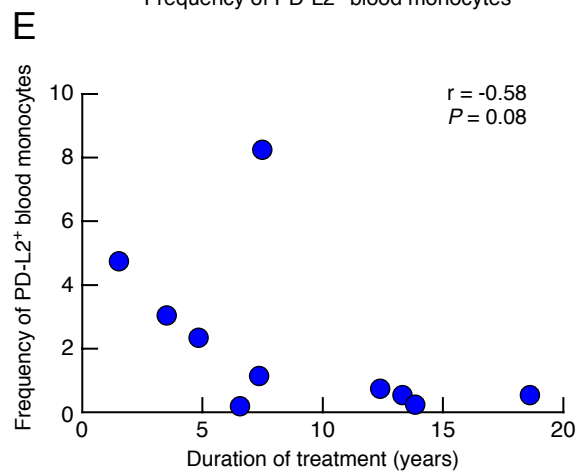

Supplement: S4 Fig — Correlation between the levels of HIV viral load and the frequencies of PD-L1+ (A), PD-L2+ (B) and CD155+ (C) blood monocytes in viremic HIV-infected patients (N = 10) and between the frequencies of PD-L1+ (D), PD-L2+ (E) blood monocytes and duration of antiretroviral therapy (years) in treated HIV-infected patients (N = 10). Grey symbols correspond to HIV-1 viremic individuals (A-C) and blue symbols correspond to HIV-infected aviremic ART treated individuals (D-E). Statistical significance (P values) was obtained using Spearman rank test for correlations. (PDF) [file ppat.1007918.s004.pdf]

# Supplemental Figure 6

A

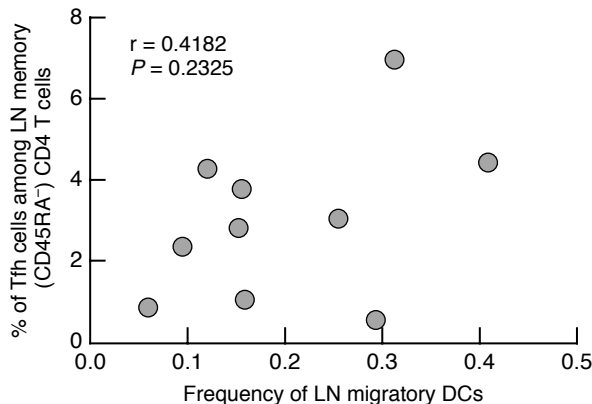

B

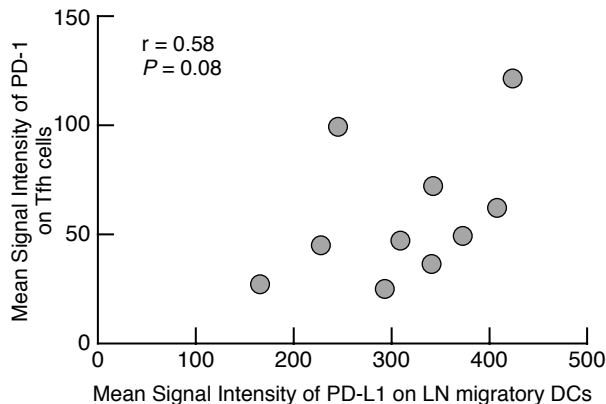

C

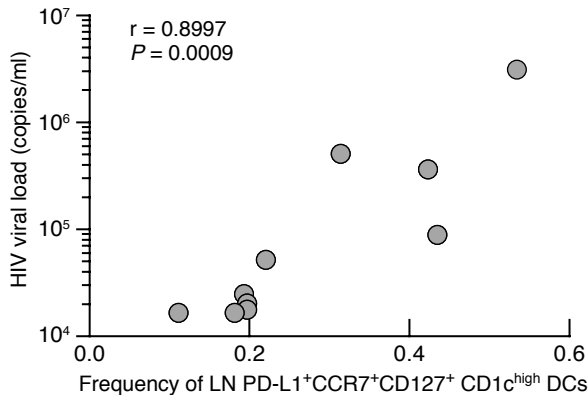

D

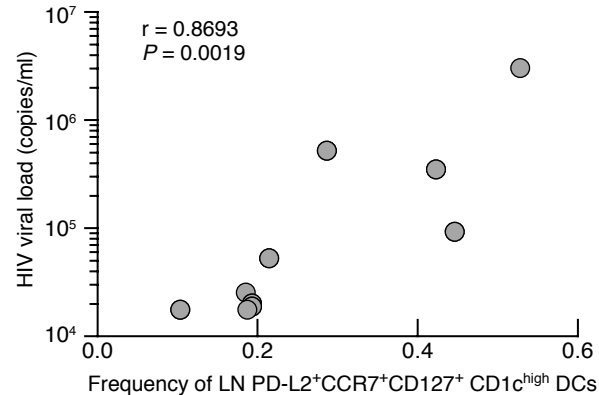

Supplement: S6 Fig — Correlation between percentage of Tfh cells and frequencies of LN migratory DCs (A) and between mean signal intensity (MFI) of PD-1 on Tfh cells and mean signal intensity (MFI) of PD-L1 on LN migratory DCs (B) in untreated viremic HIV-infected individuals (N = 10). (C) Correlation between the levels of HIV viral load and the frequencies of LN PD-L1+ migratory DCs in viremic HIV-infected individuals (N = 10). (D) Correlation between the levels of HIV viral load and the frequencies of LN PD-L2+ migratory DCs in viremic HIVinfected individuals (N = 10). Grey symbols correspond to HIV-1 viremic individuals. Statistical significance (P values) was obtained using Spearman rank test for correlations. (PDF) [file ppat.1007918.s006.pdf]
