# Supplementary material for: Lymph node migratory dendritic cells modulate HIV-1 transcription through PD-1 engagement
Source: PLoS Pathog. 2019 Jul 22;15(7):e1007918. doi: 10.1371/journal.ppat.1007918 (PMC6675123; doi:10.1371/journal.ppat.1007918)
Supplement: S5 Fig — Cumulative data of proportion of PD-L1+ (A), PD-L2+ (B) and CD155+ (C) DCs among LN HLA-DR+CD1chighCCR7+CD127+ (referred to as “DP”) and LN HLADR+CD1chighCCR7-CD127- (referred to as “DN”) DCs of HIV-uninfected (N = 7), viremic (N = 10) and aviremic ART treated HIV-infected individuals (N = 10). HIV-uninfected individuals are represented in circles, HIV viremics in triangles and HIV-infected ART treated individuals are represented in squares. “DP” and “DN” are color-coded. Red bars correspond to mean ± SEM (A-C). Red stars indicate statistical significance (* = P<0.05) (A-C). Statistical significance (P values) was obtained using one-way ANOVA (Kruskal-Wallis test) followed by Wilcoxon Matched-pairs two-tailed Signed Rank test. (PDF) [file ppat.1007918.s005.pdf]

Supplemental Figure 5

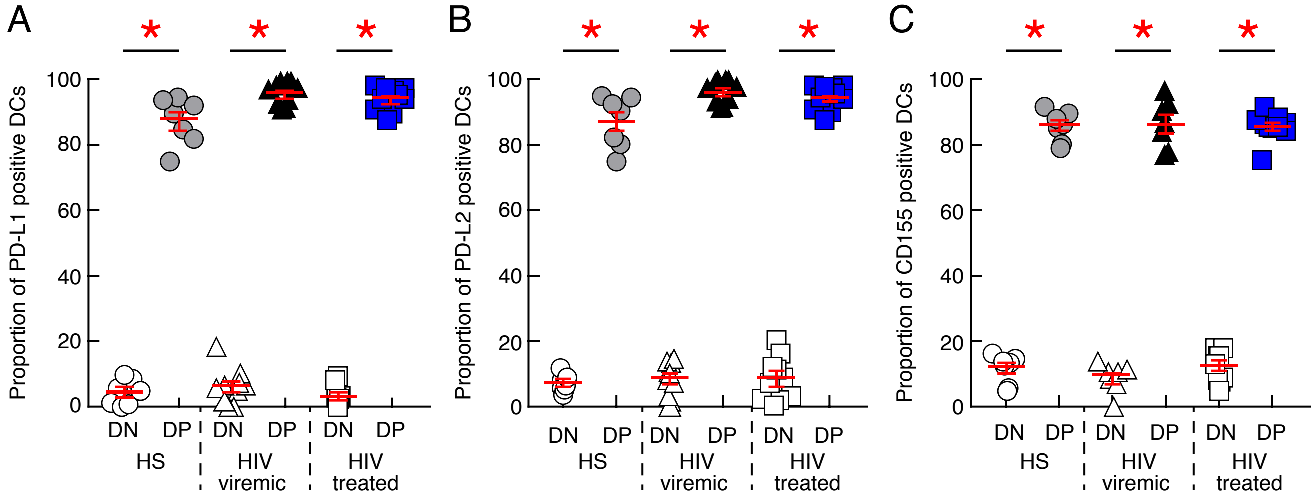

DN: HLA-DR^{+}CD1c^{hi}CCR7^{-}CD127^{-} LN DCs

DP: HLA-DR^{+}CD1c^{hi}CCR7^{+}CD127^{+} LN DCs
